# The nitrate-inducible NAC transcription factor *NAC056* controls nitrate assimilation and promotes lateral root growth in *Arabidopsis thaliana*

**DOI:** 10.1371/journal.pgen.1010090

**Published:** 2022-03-09

**Authors:** Peipei Xu, Wei Ma, Jinbo Hu, Weiming Cai

**Affiliations:** 1 Laboratory of Photosynthesis and Environment, CAS Center for Excellence in Molecular Plant Sciences, Shanghai Institute of Plant Physiology and Ecology, Chinese Academy of Sciences, Shanghai, China; 2 University of Chinese Academy of Sciences, Beijing, China; Peking University, CHINA

## Abstract

Nitrate can affect many aspects of plant growth and development, such as promoting root growth and inhibiting the synthesis of secondary metabolites. However, the mechanisms underlying such effects and how plants can integrate nitrate signals and root growth needs further exploration. Here, we identified a nitrate-inducible NAC family transcription factor (TF) NAC056 which promoted both nitrate assimilation and root growth in *Arabidopsis*. NAC056 is a nuclear-localized transcription activator, which is predominantly expressed in the root system and hypocotyl. Using the yeast one-hybrid assay, we identified the NAC056-specific binding sequence (NAC56BM), T [T/G/A] NCTTG. We further showed that the *nac056* mutant compromised root growth. *NAC056* overexpression promotes LR Initiation and nitrate deficiency tolerance. Using RNA sequencing analysis and in vitro biochemical experiment, we found *NAC056* regulated the expression of genes required for NO_3_^−^ assimilation, directly targeting the key nitrate assimilation gene *NIA1*. In addition, mutation of *NIA1* suppresses LR development and nitrate deficiency tolerance in the 35S::*NAC056* transgenic plants. Therefore, *NAC056* mediates the response of plants to environmental nitrate signals to promote root growth in Arabidopsis.

## Author summary

Nitrogen is not only a major nutrient element in plants, but also an important signal molecule for plant growth and metabolism. However, the underlying molecular mechanism of nitrate signal transduction, leading to root development response, is still unclear. In this study, by screening the subfamily III NAC transcription factors, we found the potential function of a new NAC family gene *NAC056*. The *nac056* mutant compromised root growth. *NAC056* overexpression promoted lateral root initiation and tolerance of nitrate deficiency. *NAC056* regulated the expression of genes required for NO_3_^−^ assimilation, directly targeting the key nitrate assimilation gene *NIA1*. In a high-nitrate environment, relatively high levels of *NAC056* expression promoted nitrate assimilation, further enhancing root growth. These findings reveal an important role for the nitrate-inducible *NAC056* gene in promoting nitrate assimilation and root growth in *Arabidopsis*.

## Introduction

A well-developed plant root system is critical for anchoring the plant and for absorption of nutrients and water from the soil [1]. The dicotyledonous plant root system is composed of the primary root (PR) and the lateral root (LR) system. The LR system of the plant accounts for the majority of the root system architecture (RSA) [2]. The RSA is the overall spatial arrangement of various parts of the root system, enabling plants to obtain sufficient resources from the soil. In *Arabidopsis*, LRs begin at the initials, cells that are primed in the basal meristem and activated by the initiation of xylem pole pericycle cells, and which then form a single-layer lateral root primordium (LRP) through asymmetric division. These cells are produced from PR and further divide into lateral root primordia through cell separation [3,4]. Recent studies have shown that LR development in *Arabidopsis* is mainly regulated by auxins, and that auxin signals can regulate LRs *via* two regulation modules, namely *IAA12–ARF5* and *IAA14–ARF7/19* [5–7].

The rate at which individual elements of the root system develop and grow can be determined by their response to environmental signals, including changes in nutrient concentrations, water, etc. [2,3]. Nitrate is the main form of nitrogen in the biosphere, and nitrate plays a vital role in plant root growth and development. Nitrate is also a signal molecule that controls downstream target genes. However, the underlying molecular mechanism of nitrate signal transduction leading to root development response is still unclear. In the past few years, the roles of several nitrate regulatory genes involved in major nitrate functions have been described. One of the key regulatory factors is nitrate transporter 1.1 (NRT1.1), which is a nitrate sensor [8]. Using systems biology methods, genes encoding the transcription factors TGACG SEQUENCE-SPECIFIC BINDING protein 1 (TGA1), AUXIN SIGNALING F-BOX 3 (AFB3), ARABIDOPSIS NAC DOMAIN-CONTAINING protein 79 (NAC4), and OBF BINDING PROTEIN 4 (OBP4) were identified as nitrate regulatory factors involved in nitrate signal transduction [8–10]. The MADS family transcription factors (ANR1), lateral root boundary domain (LBD), and NIN-like proteins (NLP7) regulate the growth of LR in response to nitrate (NO_3_^−^) availability. Numerous studies have also shown that NO_3_^−^ regulation of RSA entails an overlap between NO_3_^−^ and auxin signaling pathways [11,12].

NAC proteins make up one of the largest transcription factor (TF) families in plants. More than one hundred members of the *NAC* gene family exist in the *Arabidopsis* genome [13]. Recent research has revealed that many of the *NAC* members regulate *Arabidopsis* plant growth and environmental stress responses. Knock-out of the *Arabidopsis AtAF1* gene or its rice homolog *OsNAC6* reduced resistance of the plant to the respective powdery mildew fungus [14,15]. *NAC019* and *NAC055* are responsible for tolerance of both nutrient deficiency and pathogen resistance [16]. A set of *NAC* genes, *CUC1*, *CUC2*, *NAC056*, *ORE1/ANAC092*, *NAC4* and *NAC5*, are targets of the microRNA (miRNA) miRNA164, and these *NAC* genes are leaf senescence regulators [17]. *NTL6* (*NAC with Transmembrane Motif-Like 6*) plays a role in transducing freezing stress signals [18]. In the absence of water, cleavage of the transmembrane domain (TMD) allowed the transfer of NTL6 into the nucleus [19]. *Arabidopsis* NTL4 promotes reactive oxygen species (ROS) production during drought-induced leaf senescence [20].

In addition to the role of NAC TFs in regulation of plant growth and environmental stress responses, studies have shown that some NAC TFs affect nitrate metabolism and root growth. The nitrate-induction of transcription factors *NAC4* and *OBP4* required the existence of auxin receptor *AFB3*; similar to the *afb3* mutant, a *nac4* mutant was less sensitive to nitrate-stimulated LR initiation. The expression of *OBP4* was significantly reduced in *nac4* mutants [21]. Therefore, *AFB3* acts upstream of *NAC4* and *OBP4* to regulate the LR density. *OsNAC2* integrates upstream auxin and cytokinin metabolic and signaling to control root development in rice. Meanwhile, *OsNAC2* negatively regulates root formation via depressing *OsCDK* and *OsCRL* pathways [22]. *TaNAC2-5A-*overexpressing transgenic wheat lines had higher grain yield and higher nitrogen accumulation in aerial parts and allocated more nitrogen in grains in a field experiment. So, *TaNAC2-5A* is involved in nitrate signaling and it is an exciting gene resource for breeding crops with more efficient use of fertilizer [23]. Further identification of the target genes of these NAC regulatory factors, through RNA-sequencing and other molecular biology strategies, will help us to gain insights into the function of NAC TFs.

Previous studies have reported that many NAC family TFs are involved in the regulation of root development [13,15]. Our goal in the current study is to systematically reveal whether NAC TFs are involved in LR initiation and to corresponding external stress responses, such as nitrate responses. Here, we used the 90° gravitropic stimulus to synchronize LR initiation occurring on the outer surface of the curved root. By quantitatively analyzing the transcript abundance of each subgroup III of the NAC family member in *Arabidopsis* LRPs at different developmental stages, we identified possible functions of a TF member of a novel *NAC* gene family, *NAC056*. Expression of the *NAC056* gene was shown to be nitrate inducible and to play a key role in regulating nitrate assimilation and promoting root growth. Using RNA sequencing analysis and *in-vitro* biochemical analyses, we found that *NAC056* regulated the expression of genes required for NO_3_^−^ assimilation, directly targeting the key nitrate assimilation gene *NIA1*. Therefore, *NAC056* mediates the response of plants to environmental nitrate signals to promote root growth in *Arabidopsis Thaliana*.

## Results

### Expression of subgroup III *NAC* family genes in LR primordia

More than 100 genes in each of the *Arabidopsis* and rice genomes encode NAC family TFs [15,18]. Each NAC has a conserved N-terminal DNA-binding domain, which is consistent with its TF function [15]. NAC transcription factors are reported to be involved in plant response to environmental stress, and plant root development is closely related to plant response to environmental stress. In order to reveal the regulators of LR growth, we screened each member belonging to subgroup III of the *NAC* family (*NAC* III subfamily) in *Arabidopsis* (S1 Fig). The reverse transcription-quantitative PCR (RT-qPCR) method was used to quantitatively analyze the transcript abundance of each *NAC* member in *Arabidopsis* LRPs at different developmental stages. Previous work had indicated that some *NAC* family members were functionally redundant, whereas other *NAC* members played unique roles. Since many subgroup III *NAC* genes have root expression patterns [15–18], we studied whether *NAC* gene expression is related to the development of LRs and whether it responds to environmental stimuli.

**Fig 1:**
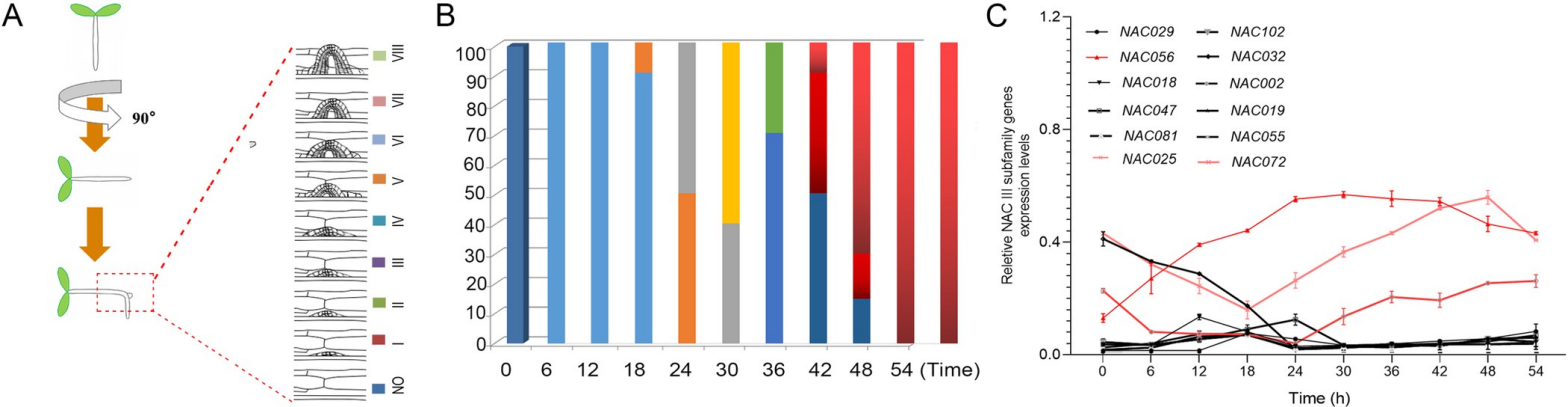
Transcriptomic analysis of subclass III *NAC* genes during lateral root (LR) initiation. B. After gravitropic stimulation to induce the synchronized initiation of lateral root primordium (LRP) at the surface of bending roots, LR primordium stages from I to VIII were assessed every 6 h between 0 and 54 h after gravitropic stimulation and are represented here as a percentage of the total number of observed LRPs at each time point. At least 90 LRPs were observed at each time point. C. Expression patterns of all subclass III *NAC* genes at each time point during LR initiation were measured every 6 h from 6 to 54 h post gravitropic induction (pgi). Bending roots of a population of 5-day-old seedlings were micro-dissected at each of ten time points, which were then used for RNA extraction (approximately 200 seedlings per time point for each of three independent replicates). Expression patterns of all subclass III *NAC* genes are shown. Each data point represents the mean ± SE (n = 3).

Gravitropic stimulation promotes the formation of LRs [4]. Through the 90° gravitropic stimulus experiment, the time of LR initiation occurring on the outer surface of the curved root becomes highly synchronized. At 12 h after the gravitropic stimulus, LRP was observed in stage 1; each subsequent LRP stage was observed at each subsequent 6-h period until LRs emerged at 48 h (Fig 1A and 1B). We checked the expression of each *NAC* III subfamily gene at each LR developmental stage (Fig 1C). Five-day-old *Arabidopsis* Col-0 plants were used in our 90° gravity stimulation assay. To verify the results, we assessed the transcriptional level of *LBD16*, a known LR regulatory gene, the expression of which was significantly induced at the reported time point (S2 Fig) [24,25]. The transcript profiles of the *NAC* III subfamily genes demonstrated that the expression levels of *NAC056*, *NAC025*, *NAC072* genes were significantly up-regulated during LR development (Fig 1C).

### *NAC056* mutation reduced LR density

**Fig 2:**
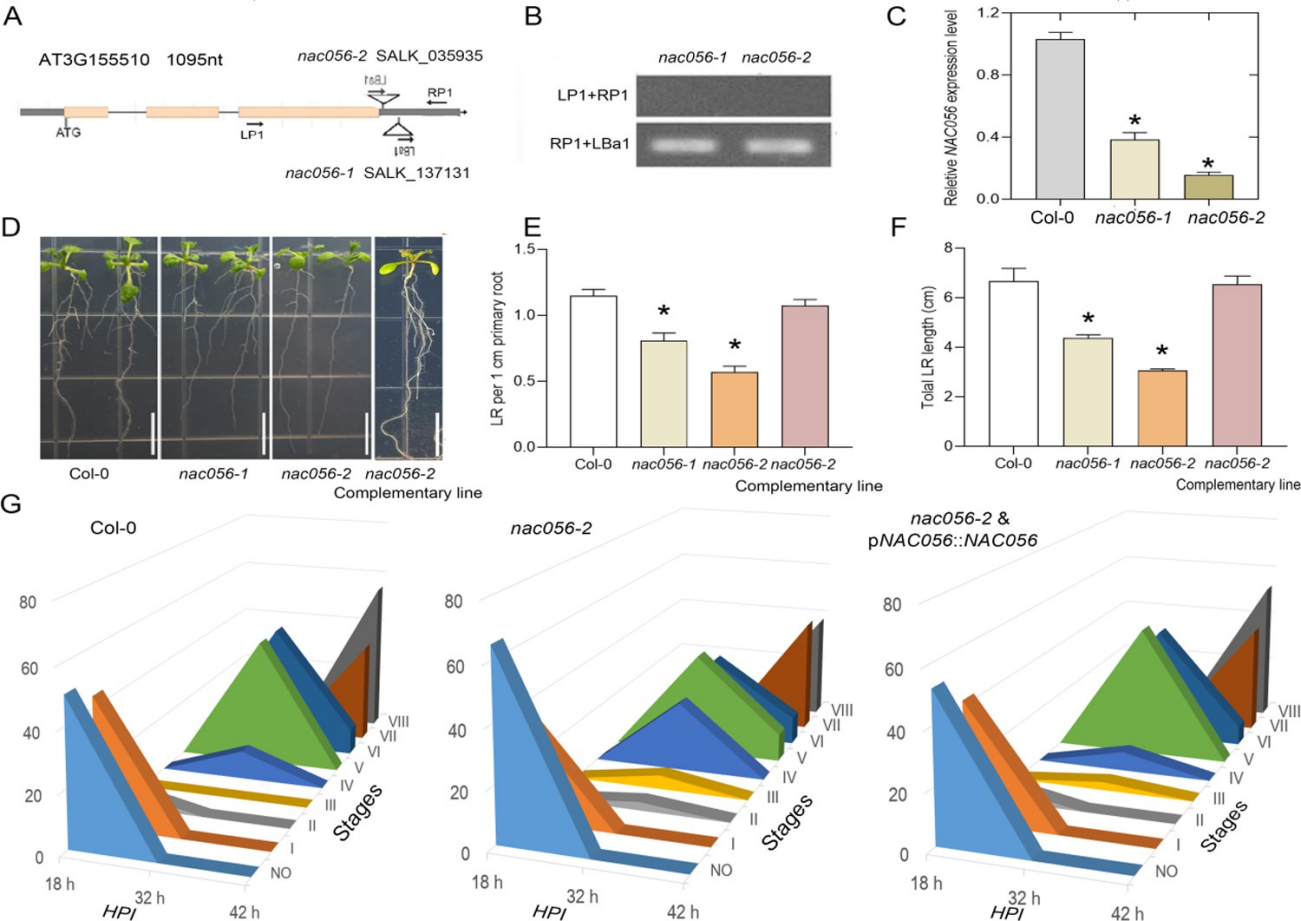
Altered root development in the *nac056* mutant. A. The genomic organization of the *NAC056* gene. The yellow boxes show the positions and sizes of the exons. The triangle indicates the site of a T-DNA insertion. The genomic sequence used to complement the *nac056* mutation is indicated. B. Identification of homozygous *NAC056* mutants. C. The mRNA abundance of the *NAC056* gene in the roots was measured in *nac056* mutant backgrounds, using reverse transcription-quantitative PCR (RT-qPCR). D. Root phenotypes of wild-type (WT) plants, the *nac056* mutants, and complemented mutant lines. The plants were grown vertically on medium for 8 d. Bar indicates 1 cm. (E) LR density and (F) total LR length analysis in the Col-0, *nac056* mutant, and *nac056* complemented mutant lines. The error bars denote the standard errors (SEs) (n = 6–8). G. Phenotypic analysis of LR emergence (LRE) was achieved by synchronizing LR formation with gravitropic stimulus for 18, 32 or 42 h. Compared with the WT, the *nac056-2* mutant showed delayed LRE. Mutant plants transformed with the full-length *NAC056* genomic fragment exhibited a WT LRE phenotype for LR induction. The data are shown as percentages, and the error bars represent SEs (n = 10–16). At least 100 total LRPs were observed for each plant. Each data point represents the mean ± SE (n = 4). *p < 0.05 by Student’s *t*-test.

Under gravitropic stimulation, the gene expression profiles of some members of the *NAC* subgroup III were affected. Therefore, we attempted to better understand the role of selected subgroup III *NAC* member genes. In order to further identify the functions of these genes, we obtained the corresponding homozygous T-DNA insertion lines [26]. By observing the root phenotypes, we identified root growth defects associated with some of the insertion mutants of selected subgroup III *NAC* genes. Lines SALK_137131 (named *nac056-1*) and SALK_035935 (named *nac056-2*) were shown to have inserts in the 3’-UTR (untranslated region) of *NAC056* (Fig 2A). After careful observation, we found LR root growth defects in *nac056* mutants. No significant changes in root phenotypes were found in *nac025* and *nac072* mutants (S3A–S3C Fig). Therefore, we focused on *NAC056*. In the *nac056* mutant alleles, insertion sites were in the 3’-UTR region (Fig 2A and 2B). *NAC056* transcriptional levels in mutant lines were identified by RT-qPCR and quantitative real-time PCR (qPCR) analysis. This led to (1) a significant reduction in the RT-qPCR amplification of *NAC056* upstream of the insertion site (Fig 2C) and (2) no detectable amplification of the RT-PCR product across the entire *NAC056* coding sequence (CDS) in a *nac056-2* mutation background (S3D Fig). We came to the conclusion that all the *nac056* mutants were either strong reduction-of-function or null loss-of-function alleles.

Under experimental growth conditions, the *nac056* mutations reduced the development of LRs and affected the development of the primary root (Fig 2D–2F). In addition, we complemented the *nac056-2* mutant root phenotype with the full-length genomic DNA of *NAC056*, with the complemented mutant plants exhibiting normal root growth (Fig 2G). Then, Col-0 (wild type, WT), mutant and complementary transgenic lines were tested in gravitropic stimulation assays, and the number of LRPs present at 18-, 32-, and 42-h post gravitropic induction (pgi) was counted. The Col-0 plants accumulated stages I to III LRPs at 18-h post-gravitropic stimulation, and stages VI to VIII LRPs at 42-h of post gravitropic stimulation. However, compared with the wild type, the *nac056-2* mutant showed a lower proportion of LRPs at stages V–VI at 32-h pgi and a lower accumulation of LRPs at phases VII–VIII at 42-h pgi (Fig 2G). The LR emergence rate of the complemented mutant plants was consistent with that of the Col-0 plants (Fig 2G). The above results showed that *NAC056* positively regulated LR development. Subsequently, we investigated the underlying molecular mechanisms.

### *NAC056* expression in the LRs and its transcript abundance is induced by nitrate in roots

**Fig 3:**
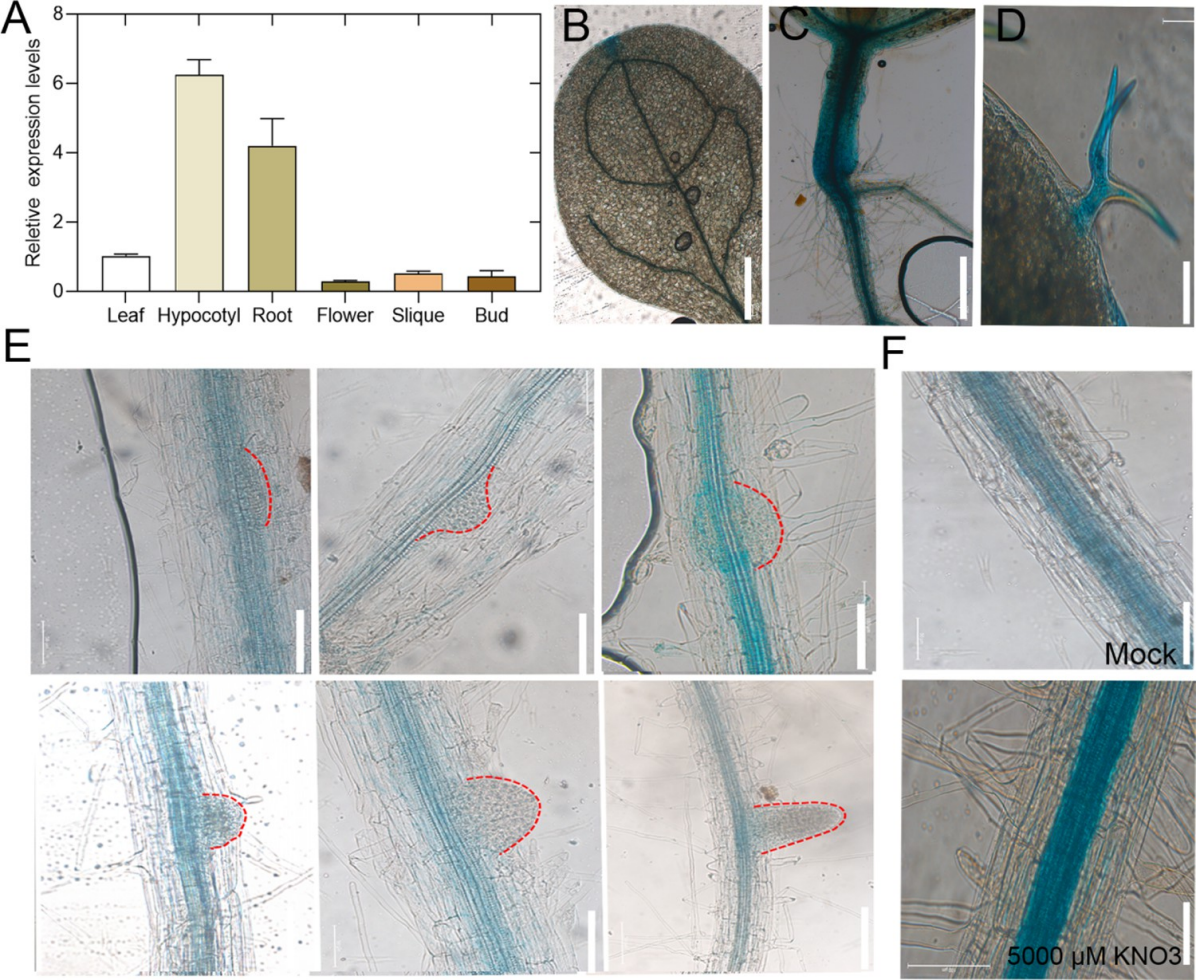
Analysis of proNAC056::GUS transgenic lines during LR development. A. Relative *NAC056* expression level in young fully expanded leaf, root, hypocotyl, silique, flower, or flower bud. Three independent replicate experiments were conducted. Values are given as mean ± standard error (SE), n = 3. (B-D). GUS staining in proNAC056::GUS *Arabidopsis* leaf, root, hypocotyl and trichrome. E. GUS staining in proNAC056::GUS *Arabidopsis* LRs at various developmental stages. F. The seedlings were grown on medium supplemented with 5,000 μM KNO_3_ for 4 h. Images of the GUS signals in roots were obtained, and representative images are shown. The white bar indicates 50 μm.

Given that *NAC056* expression was up-regulated during LR emergence and the observation that root growth in *nac056* mutants was defective, the expression level of *NAC056* in various organs (seedlings, shoots, rosette leaves, buds, hypocotyls, roots, and flowers) was determined. *NAC056* was highly expressed in hypocotyls and the root system (Fig 3A). In addition, we constructed proNAC056::GUS transgenic plants driven by a native 2.6 kb promoter. We obtained many transgenic lines and used one of them to check the GUS signal in the various stages of LR primordium development. We could observe GUS signals from LR initiation to the elongation stages of LR development and primary root in these lines. *NAC056* was expressed throughout LR development (Fig 3B–3E).

**Fig 4:**
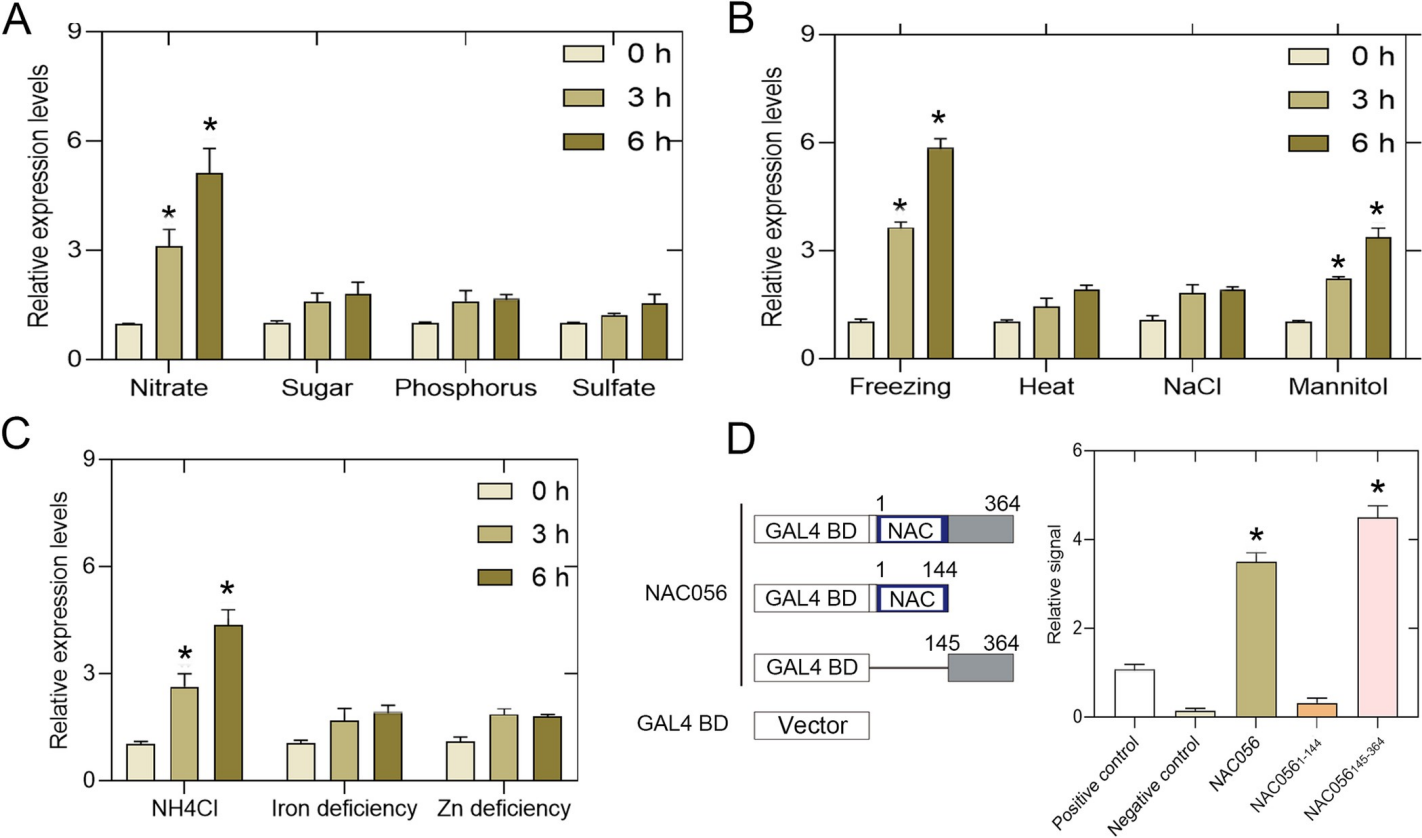
Analysis of *NAC056* expression in response to environmental stress and transactivation activity analysis. A-C. Effects were determined in response to various abiotic stress treatments (100 mM NaCl, freezing (−4°C), heat (28°C), or 200 mM mannitol) or nutrition (6% sugar, 1 mM KNO_3_, 1 mM KH_2_PO_3_, 0.5 mM NH_4_Cl, 0.2 mM sulfate, 2 μM FeSO_4_.7H_2_O, or 1 μM ZnCl_2_) on *NAC056* expression levels in *Arabidopsis* roots. *AtACT2* was used as an internal control. Values are given as mean ± SD, n = 3. *p < 0.05 by Student’s *t*-test. D. Transactivation activity assays of *NAC056* in *Arabidopsis* protoplasts. The full-length open-reading frame (ORF) of *NAC056* and its deletion constructs were used in the assay. NAC represents the DNA-binding domains of *NAC056*. The negative control was the effector vector without the gene inserts. The effector vector used is shown in the left panel. The *GAL4* transient expression assays were performed using *Arabidopsis* protoplasts, shown in the right panel. Three independent experiments were conducted. Values are given as mean ± standard error (SE), n = 3. *p < 0.05 by Student’s *t*-test.

In order to evaluate whether environmental factors affected the expression of *NAC056*, we checked the effects of various abiotic stress treatments (freezing, osmotic stress (mannitol), heat, or salt) and nutrient deficiency treatments (nitrate, sugar, phosphorus, sulfate, NH_4_Cl, Zn, or iron deficiencies) on the level of *NAC056* mRNA in roots. Among these treatments, nitrate treatment significantly promoted the development of LR, and also increased *NAC056* expression level (Figs 4A, S4). The response of the *NAC056* transcript abundance to NO_3_^−^ was specific to this anion, because sugar, phosphate and sulfate did not result in significant changes in transcription. Ammonium (NH_4_^+^) also induced expression of the gene, but the degree of induction was weaker than nitrate (Fig 4C). Freezing for 6 h increased *NAC056* expression more than 5-fold, whereas mannitol treatment increased expression of this gene only 3-fold, with the other abiotic stress treatments having no significant effects on expression (Fig 4B). The nitrate reductase (NR)-deficient *nia1nia2* mutant grown on NO_3_^−^-free medium exhibited the induction of *NAC056* gene expression after KNO_3_ re-addition, which indicates that the signal from NO_3_^−^ itself has triggered the increase in *NAC056* transcript abundance (S5 Fig). Additional transcription profile analysis and reporter gene studies indicate that *NAC056* was expressed after nitrate treatment. Therefore, we concluded that *NAC056* gene expression was induced by exposure to nitrate.

### The C-terminal domain of *NAC056* exhibited transactivation activity

The *NAC056* gene encodes a 364-amino-acid (aa) protein. The N-terminus contains five conserved homology blocks that characterize the NAC family. The NAC domain was detected between 17 aa and 144 aa of NAC056. The divergent C-terminus of NAC056 has no obvious homology with other family members. Due to the fact that NAC056 contains both a nuclear localization signal (NLS) (between 287 aa-315 aa) and a DNA-binding domain suggests that NAC056 could be a functional TF. To verify whether NAC056 is a functional transcription factor requires evidence for nuclear localization. The *NAC056-*GFP construct was transformed into tobacco leaves. Then, the green fluorescent protein (GFP) signal was screened for, with the signal being localized in the nucleus of tobacco leaf cells (S6 Fig). We further removed the NLS sequence of NAC056 protein, and the truncated NAC056 protein could not be fully located in the nucleus. Therefore, the NAC056 protein nucleus localization relies on this NLS signal (S6 Fig). In addition, to identify the ability of NAC056 to activate transcription *in vivo*, we used *Arabidopsis* protoplasts as the study system and luciferase as the reporter enzyme. Here, the fusion of NAC056 and the GAL4 DNA-binding domain was tested for the ability to activate the transcription of the GAL4 upstream activation sequence (UAS). The result confirmed that the intact NAC056 protein showed activation ability, with the C-terminal fusion protein also being an effective activator (Fig 4D). These results, together with the presence of the NLS, which localizes proteins to the nucleus, confirm the model that NAC056 is a functional transcriptional activator.

### The *nac056* mutants were defective in nitrate-promoted root growth

**Fig 5:**
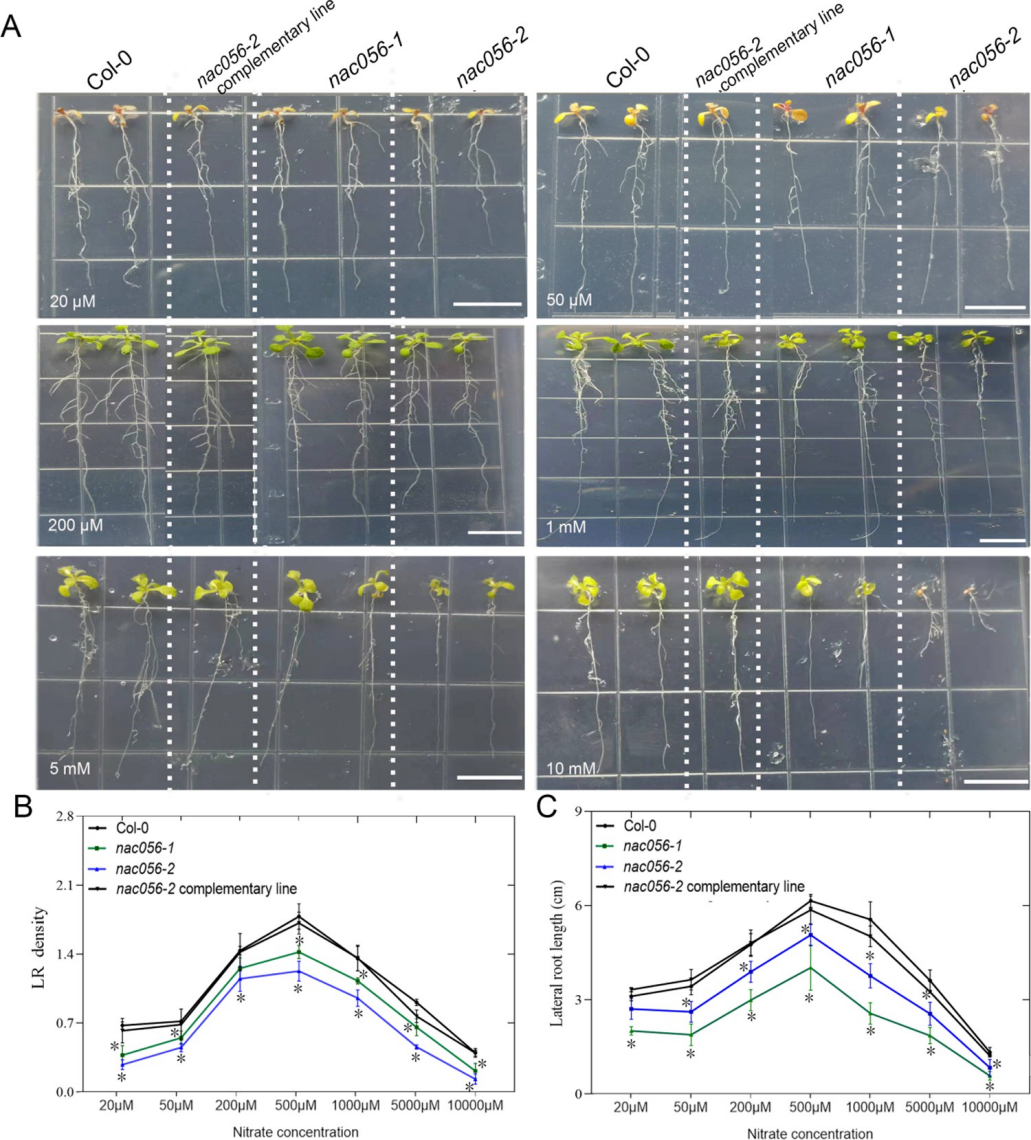
Nitrate-promoted root growth analysis in Col-0 and the *nac056* mutants. A. Effects of various concentrations of nitrate (20 μM, 50 μM, 200 μM, 500 μM, 1 mM, 5 mM or 10 mM) on root growth. WT, *nac056* mutants, and the complemented mutant line were grown vertically in medium supplemented with different nitrate concentrations for eight days. B. LR density; C. lateral root length were quantified. LR density was calculated as the LR number per 1 cm of primary root. *indicates significant difference from Col-0 (*p* <0.05) by Student’s *t*-test. Data are mean ± standard error (SE) (n = 4). The black bars indicate 1.2 cm.

*Arabidopsis* plants, in which the roots were exposed to different concentrations of nitrate, were assayed. We analyzed the response of 8-day-old plants of Col-0, *nac056* mutants, and the complemented mutant plants to growth on medium containing various nitrate concentrations (Figs 5 and S7). The effect of nitrate on root growth is concentration dependent. Nitrate concentration can promote the growth of LRs, but too low or too high a nitrate level can inhibit the growth of LRs. We studied the root development of plants of Col-0 wild type, *nac056* mutants and complemented mutants on medium supplemented with concentrations of 20 μM, 50 μM, 200 μM, 500 μM, 1 mM, 5 mM, or 10 mM nitrate. The results indicated that, under conditions of nitrogen deficiency (20 μM nitrate), both Col-0 and *nac056* mutants showed the phenotype of nitrogen deficiency, with strong inhibition of the initiation of LRs. In addition, the LR density of *nac056* mutants was significantly less than that of Col-0 wild type (Fig 5A and 5B). When the medium was supplemented with 200–1,000 μM nitrate, these concentrations of nitrate clearly promoted root growth of the wild type, but root growth of the *nac056* mutants was not sensitive to these nitrate concentrations. In addition, the LR density and LR length of *nac056* mutants was significantly lower than that of Col-0 (Fig 5B and 5C). Concentrations of 5–10 mM nitrate inhibited LR emergence in Col-0 and *nac056* mutants, but the inhibitory effect was greater in *nac056* mutants than in Col-0 (Fig 5B and 5C). With the increase in nitrate concentration, the slope of the LR density and LR length dose-response curves of *nac056* mutants were obviously lower than those of Col-0, but the LR density and LR length reverted to the Col-0 level in the complemented mutants. The conclusion is that the *nac056* gene mutation affects the nitrate-inducible promotion of lateral root growth.

### Genes differentially expressed in *nac056* mutant

**Fig 6:**
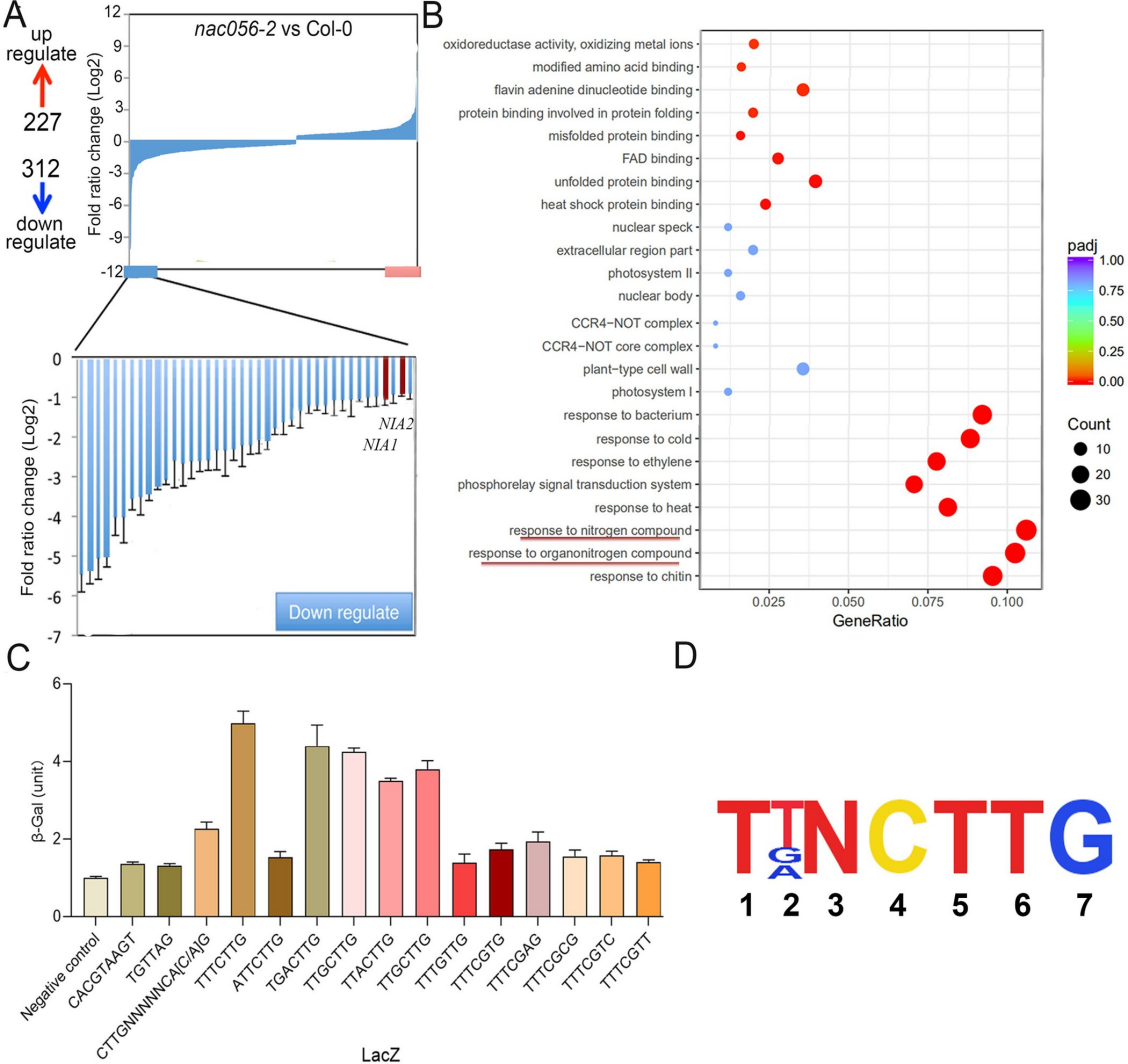
The differentially expressed genes in the *nac056-2* mutant, and *NAC056* binding-motif analysis. Relative expression levels of more than 500 genes that are induced or repressed by the *nac056* mutation based on microarray analysis. B. GO (Gene Ontology) enrichment analysis of the top pathways in the down-regulated genes between Col-0 and the *nac056-2* mutant. C. Interaction of NAC056 with the NAC056BM and its substituted sequences in yeast one-hybrid assays. The sequences of interest were fused to a promoter fragment of ACT2 (negative control) that does not contain a NAC056BM. β-Galactosidase (β-Gal) activity (1 unit = 10^4^ min^-1^ mL^-1^) was measured by liquid assays using chlorophenol red-β-D-galactopyranoside (CPRG; Roche Applied Science) according to the Yeast Protocol Handbook (Clontech). The yeast strain YM4271 was used in the assay. D. Figure indicates the core sequences of NAC056BM.

To identify the downstream genes affected by the *nac056* mutation and to reveal the underlying molecular mechanisms of *nac056*-mediated root growth defects, we used RNA sequencing (RNA-Seq) analysis to detect differentially expressed genes (DEGs) between the Col-0 wild type and the *nac056-2* mutant. The hypocotyls and roots of 11-day-old seedlings of Col-0 and *nac056-2* grown on half-strength Murashige & Skoog (MS) medium were isolated for microarray analysis (Fig 6A). We found more than 500 genes with significantly altered expression levels between the two genotypes, about 227 of which were up-regulated (*nac056*/WT >2-fold), with approximately 312 being down-regulated (*nac056*/WT < 0.5-fold) (Fig 6A). Because we had shown that NAC056 functioned as a transcriptional activator, we deduced that the target genes of NAC056 could be among the genes with down-regulated expression levels in the *nac056* mutant background. Gene Ontology (GO) enrichment analysis of all the down-regulated genes showed that terms relating to organic nitrogen metabolism, stress response, defense and oxidation /reduction, response to ethylene etc. were enriched (Fig 6B). Among the over-represented genes related to nitrate metabolism, nitrate assimilation genes *NIA1* and *NIA2*, associated with the key step of nitrate assimilation, were enriched. These genes encode the cytosolic minor isoform of nitrate reductase (NR) and exhibit nitrate reductase activity [27]. Thus, we had confirmation that these genes were affected by the *nac056* gene mutation (S8 Fig).

### *NAC056* binding-motif analysis

Earlier studies had found that the CACG core motif was the binding sequence of NAC TFs [13]. In addition, by using a combination of base sequence analysis tools and promoter-binding assays, the NAC TF binding base sequences have been determined, which indicates that each NAC TF may have a unique binding sequence [28,29]. For example, the SNAC (stress response family NAC) binds to the TTNCGT(G/A) sequence [30], whereas the *NTL8/ANAC040* (NTLBS) binding sequence is TTNCTT [31]. However, some *Arabidopsis* transmembrane motif (TMD)-containing NAC TFs specifically interact with the mitochondrial dysfunction motif (MDM) CTTGXXXXX CA[C/A]G [31], whereas the *Arabidopsis* NAC016 specifically binds to the NAC016-binding motif (NAC016BM) GATTGGAT[A/T]CA [19]. Therefore, it is possible that NAC056 binds these motifs or their variants. We used yeast one-hybrid assays to identify which motifs of candidate sequence are functional for NAC056 (Fig 6C). Ultimately, we identified the likely NAC056-DNA binding motif sequence to be T[T/G/A]NCTTG (NAC056BM) (Fig 6D). Furthermore, we conducted point mutant analysis of NAC056BM and quantified the binding ability among them. We found that the T (1st) and CTTG (4th to 7th) point mutations of NAC056BM significantly decreased the NAC056-DNA binding ability, indicating that these bases are critical for NAC056-DNA binding. But other point mutations, such as at the 5th or 6th site, did not change the binding ability significantly (Fig 6C). Interestingly, the NAC056BM shows some similarities to the mitochondrial dysfunction motif (MDM).

### *NAC056* regulates genes involved in NO_3_^−^ assimilation

**Fig 7:**
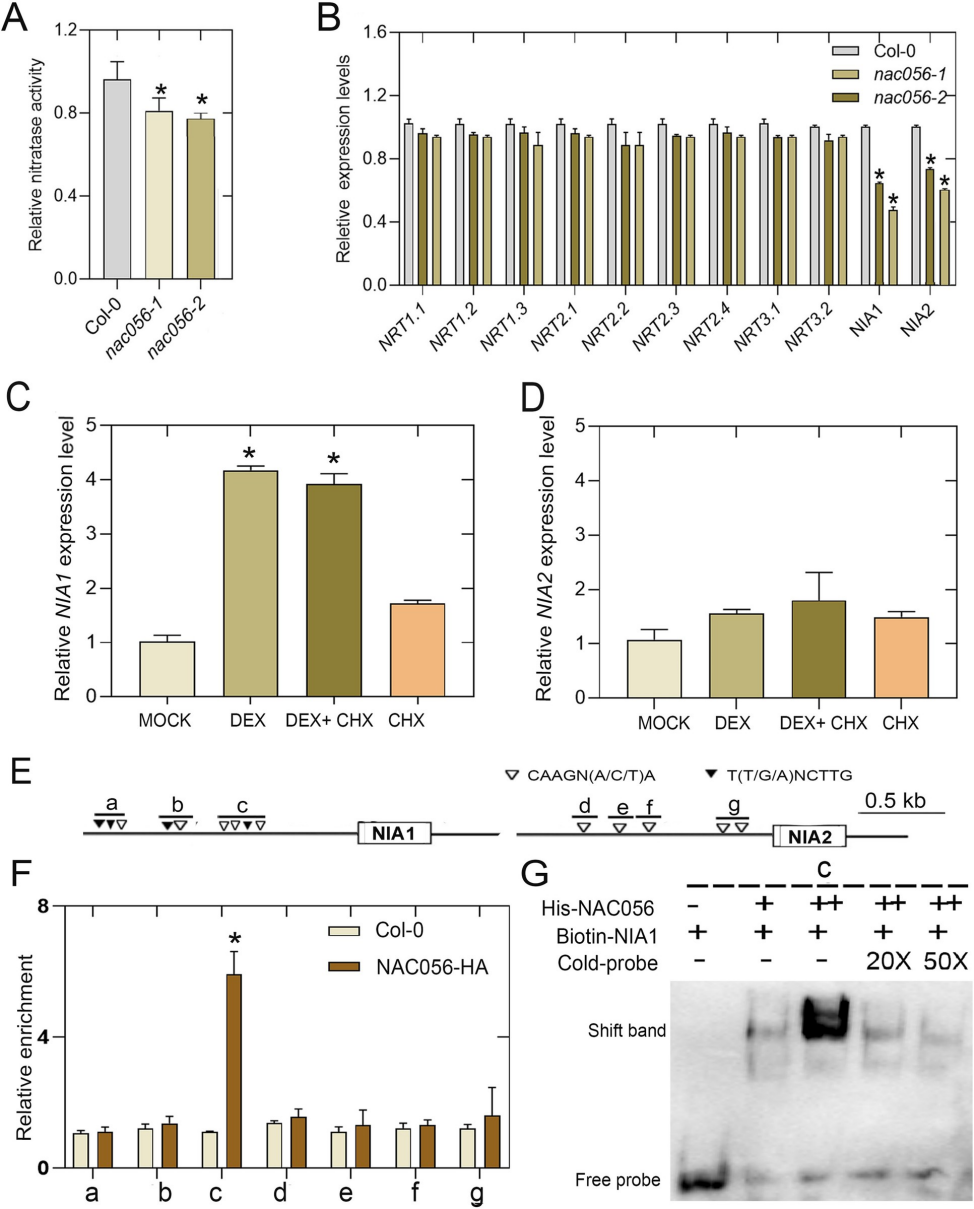
*NAC056* regulates genes involved in NO3− assimilation. A. Measurement of relative nitrate reductase activity in the roots of 11-day-old wild-type and *nac056* mutant seedlings. Values are given as mean ± standard deviation (SD), n = 3. *p < 0.05 by Student’s *t* test, in comparison with Col-0. B. RT-qPCR analysis of nitrate transport- and assimilation-related genes in Col-0 and *nac056* mutant plant roots. Values are given as mean ± SD, n = 3. *p < 0.05 by Student’s *t-*test, in comparison with Col-0. Relative expression level of (C) *NIA1* and (D) *NIA2* in 8-day-old *NAC056*GR transgenic plants treated with 30 μM DEX, 100 μM CHX, 30 μM DEX plus 100μM CHX or mock (control). The gene expression in mock-treated plants was set to 1.0. Values are given as mean ± standard deviation (SD), n = 3. *p<0.05 by Student’s *t* test. E. Schematic diagram showing the *NIA* genomic region and location of sense NAC056BM (T[T/G/A]NCTTG, closed triangles) and anti-sense NAC056BM (CAAGN [A/C/T]A, open triangles) motifs. Schematic diagram indicating the locations of NAC056 motif clusters (a to g) in the *NIA1* and *NIA2* gene promoters. F. ChIP-qPCR analysis of the ability of NAC056 to bind to the promoters of *NIA1* and *NIA2*. An anti-HA monoclonal antibody was used for DNA immunoprecipitation from three-week-old *NAC056*-HA transgenic plants. Black bars indicate the enrichment fold-changes normalized to that of *ACT2*. Values are given as mean ± SD, n = 3. *p < 0.05 by Student’s *t-*test. G. EMSA (Electrophoretic Mobility Shift Assay) showing that NAC056 binds to the promoter of *NIA1* (fragment c). Each biotin-labeled DNA fragment was incubated with His-NAC056. Competition assays for the labeled promoter sequences were performed by adding an excess of unlabeled cold probe. Two biological replicates were performed, achieving similar results. The representative results were shown.

Because the *nac056* mutants exhibited defective root growth in response to nitrate supplementation, and the DEGs between Col-0 and *nac056-2* plants were enriched with respect to the nitrate metabolism pathway, these findings imply that NAC056 functions in the transcriptional regulation of N responses. It is known that nitrate is the primary factor regulating NR induction. NR activity is also affected by many other environmental factors. To test whether nitrate assimilation was changed in *nac056* mutants, we checked the NR activity in Col-0 WT and *nac056* mutant plants in the presence and absence of freezing stress. We found that the *nac056-2* mutant reduced the relative nitrate reductase activity by 25% (Fig 7A). These results indicated that the nitrogen assimilation process regulated by *NAC056*. To reveal the underlying mechanism of NAC056 in the transcriptional control of nitrate assimilation, we identified NO_3_^−^ uptake- and reduction-related genes, including NR genes (*NIA1*, *NIA2*) and NO_3_^−^ transporter genes (*NRT1*.*1*, *NRT1*.*2*, *NRT1*.*3*, *NRT2*.*1*, *NRT2*.*2*, *NRT2*.*3*, *NRT3*.*1* and *NRT3*.*2*) and some nitrate regulatory genes. Among them, we found that *NIA1* and *NIA2* were significantly downregulated in the *nac056* mutants (Fig 7B).

Furthermore, to identify any direct relationship between *NAC056* and *NIA* genes, we quantified the transcriptional activation of *NIA* genes by *NAC056* over a time course. We used the root tissues from the plants treated with or without the synthetic steroid hormone dexamethasone (DEX) over a period of time (S9 Fig). The results showed that expression of *NIA1*, but not *NIA2*, was significantly up-regulated after 3 h induction with DEX (Fig 7C and 7D**)**. We then repeated the DEX treatment experiment in the presence and absence of the translation inhibitor cycloheximide (CHX) to inhibit protein synthesis [32]. Expression of *NIA1* was still rapidly induced by DEX treatment in the presence of the CHX treatment (Fig 7C**)**. We analyzed the possible *cis* elements that had previously been identified as binding sites for NAC056. A number of *cis* elements were found in *NIA* gene promoters (Fig 7E). We conducted chromatin-immunoprecipitation–real-time PCR (ChIP–qPCR) assays to test whether NAC056 was associated with the selected promoter regions of the *NIA* genes (Fig 7F). We then used purified NAC056 protein (S10 Fig) and the synthesized *NIA1* gene promoter region, containing clusters of NAC056BM, to carry out the Electrophoretic Mobility Shift Assay (EMSA) assay. When the NAC056 protein was added, migration of the biotin-labeled probes shifted dramatically. This band shift was reversed by adding cold-competitive probes (Fig 7G). The results indicate that NAC056 directly binds to region c but not to regions a/b of the *NIA1* promoter. Although both *NIA1* and *NIA2* are regulated by *NAC056* transcription factor, only *NIA1* is the direct downstream target gene, and the regulation of *NIA2* may be indirect or dependent on other intermediate regulators. So, NAC056 is involved in regulating NO_3_^−^ assimilation by directly targeting the *NIA1* gene.

### *NAC056* overexpression promotes LR initiation and tolerance of nitrate deficiency

**Fig 8:**
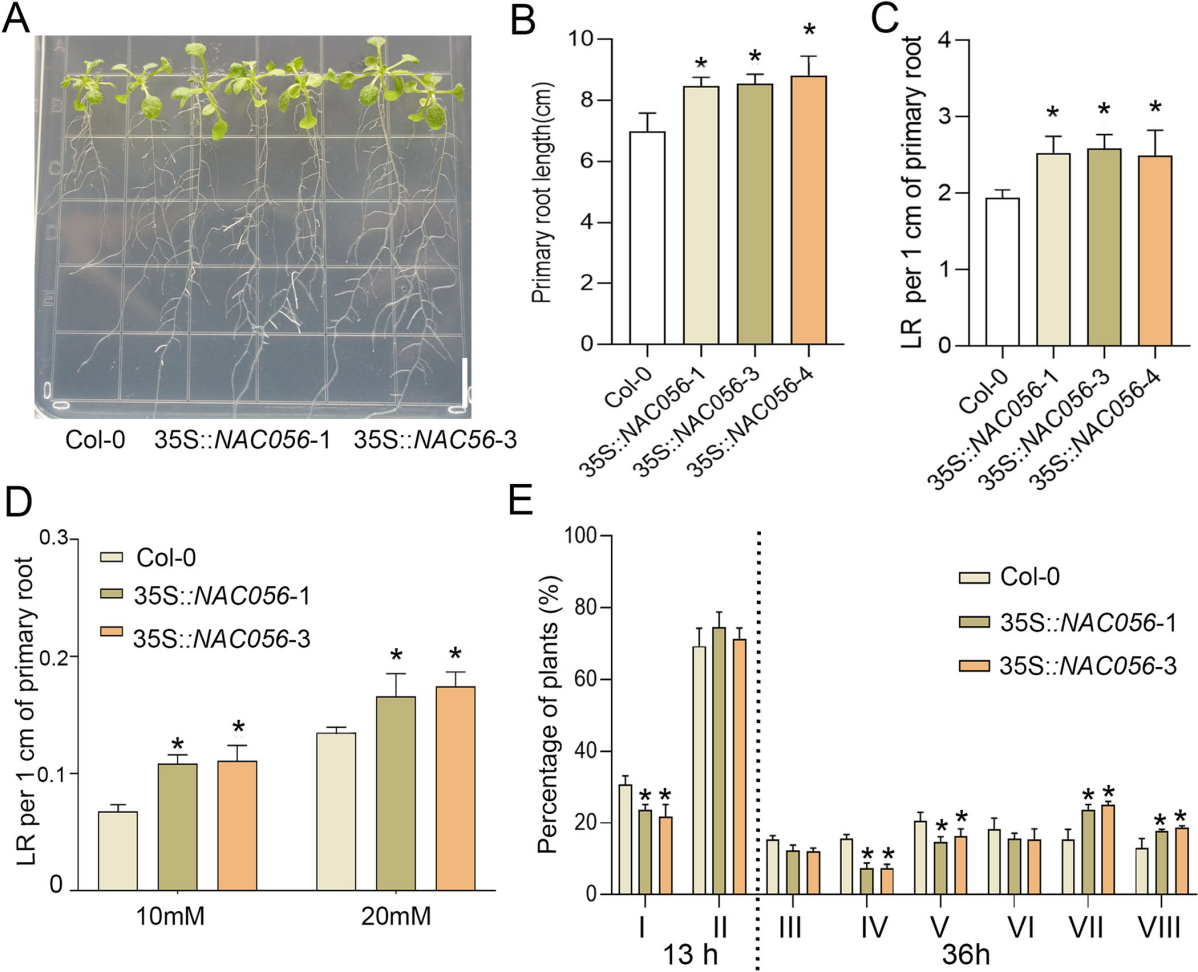
35S::*NAC056* promoted LR growth and increased low-nitrate tolerance. Root phenotype observation and analysis (B) primary root length; (C) LR density in ten days old Col-0 and *NAC056*-overexpressed lines. Values are given as mean ± standard error (SE), n = 3. *p < 0.05 by Student’s *t* test. D. Col-0 and transgenic plants were grown vertically in nitrogen- deficient medium supplemented with 10 μM or 20 μM nitrate for 9 d. Average LR density was quantified. Data points are mean ± SE. Values are given as mean ± SE, n = 3. *p < 0.05 by Student’s *t* test. E, Phenotypic analysis of LRE was achieved by synchronizing LR formation with gravitropic stimulus for 13 h and 36 h. Compared with the WT, the transgenic plants showed increased LRE. The data are shown as percentages, and the error bars represent SDs (n = 6–12). At least 90 total LRPs were observed for each sample.

Because of the defective LR growth exhibited by the *nac056* mutant, we investigated the root system architecture (RSA) in 35S::*NAC056* plants. Ten of the fourteen independent *NAC056***-** overexpressed lines we obtained had high *NAC056* expression level (S11 Fig**)**, with these overexpressed lines exhibiting markedly increased growth of LRs and primary roots (Fig 8A–8D). Because the *nac056* mutant exhibited decreased nitrate sensitivity, we investigated whether 35S::*NAC056* exhibited increased tolerance of nitrate deficiency. The results showed that the 35S::*NAC056* exhibited much greater root growth and increased LR density than Col-0 at low nitrate concentrations (10 μM and 20 μM) (Fig 8D). The Col-0 and 35S::*NAC056* plant roots were subjected to the gravitropic stimulus, and LRPs were checked at 13 h and 36 h pgi (Fig 8E). The results indicated that 35S::*NAC056* significantly promoted both LR emergence and primary root growth.

### The *nia1* mutant suppress LR development and nitrate deficiency tolerance in 35S::*NAC056* overexpressed lines

**Fig 9:**
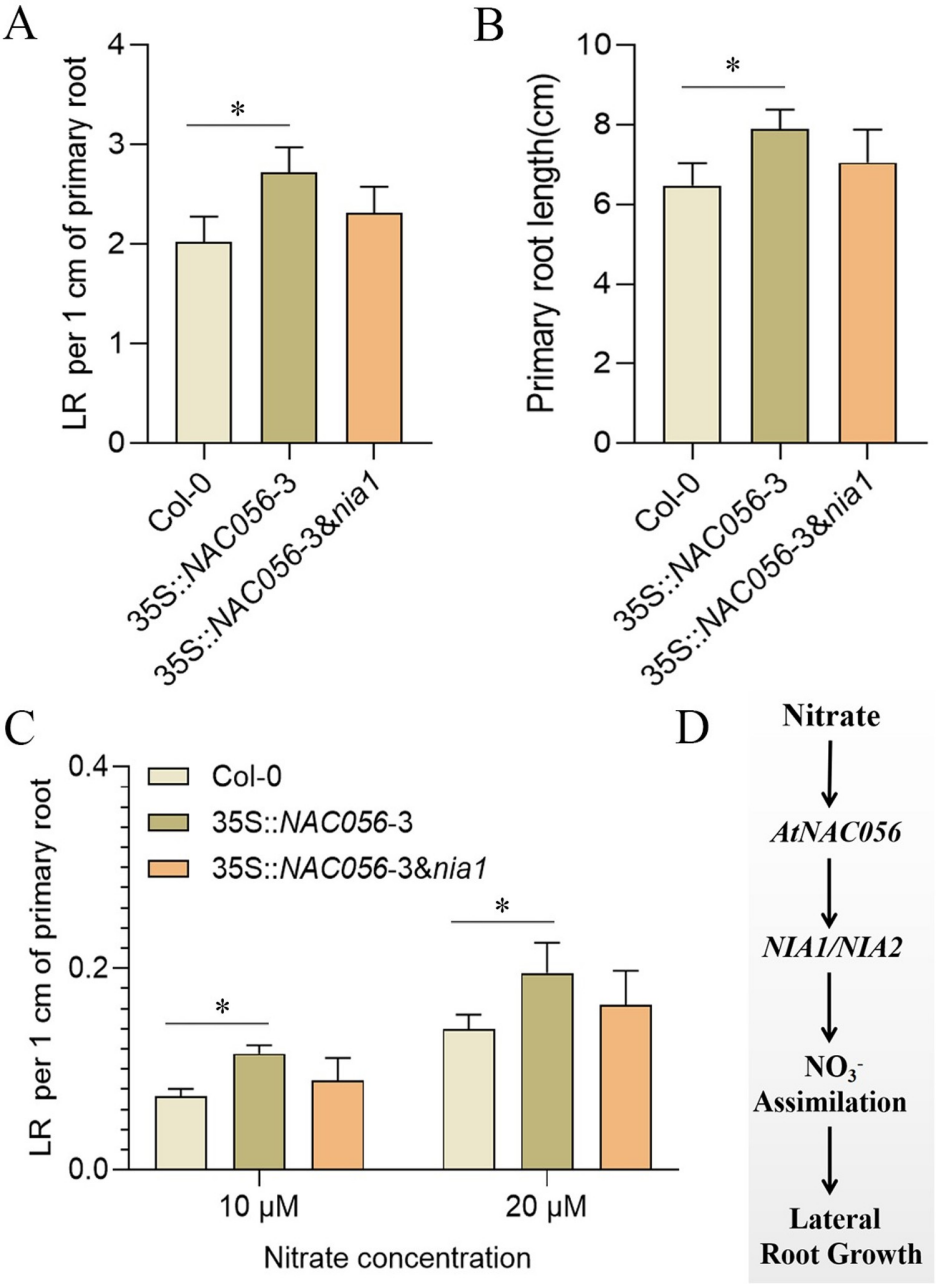
Root phenotype analysis in Col-0, 35S::*NAC056* and 35S::*NAC056 & nia1* transgenic lines and a model for *NAC056* functions. Root phenotype observation and analysis of (A) primary root length; (B) LR density in 9-day-old Col-0, 35S::*NAC056* and 35S::*NAC056 & nia1* transgenic lines. Values are given as mean ± error (SE), n = 3. *p < 0.05 by Student’s *t* test. C, Col-0 and transgenic plants were grown vertically in nitrogen-deficient medium supplemented with 10 μM or 20 μM nitrate for 8 d. Average LR density was quantified. Data points are mean ± SD. Values are given as mean ± SE, n = 3. *p < 0.05 by Student’s *t* test. D, A model is proposed for NAC family transcription factor NAC056 in the regulation of LR growth. In a high-nitrate environment, *NAC056* expression is significantly induced, directly inducing nitrate assimilation to promote root growth. Altogether, *NAC056* has an important role in promoting root growth in response to environmental nitrate availability.

Our data indicated that *NAC056* was involved in the control of nitrate reductase by targeting the *NIA1* promoter. Moreover, to determine whether promotion of lateral root growth in *NAC056* -overexpressing plants depends on *NIA1*, we obtained the *nia1* mutant. Then, a 35S::*NAC056* transgenic plant was crossed with the *nia1* mutant. We analyzed the progression of root growth in the wild-type control and in various transgenic lines. The roots of the 35S::*NAC056* & *nia1* hybrid showed decreased root growth compared with the 35S::*NAC056* plants (Fig 9A and 9B). Moreover, 35S::*NAC056* overexpression in the *nia1* mutant background further inhibited the nitrate deficiency tolerance phenotype to the wild-type control level (Fig 9C). Taken together, these findings concluded that the loss of *NIA1* function suppressed root growth in plants overexpressing *NAC056*.

## Discussion

Nitrate plays a signal regulatory role in many physiological processes, including root growth. Nutrient uptake efficiency depends on root system architecture in the soil [9], and these processes are controlled by several regulatory genes, which are, in turn, regulated by nitrate [10]. Nitrogen is not only a plant macro-nutrient but also an important signal molecule for plant growth and metabolism. However, information on the underlying molecular mechanisms is still inadequate. In this article, we screened the class III subfamily NAC TFs and found the potential functions of a nitrate-inducible member of a novel NAC TF gene family. In recent years, the functions of some plant-specific NAC TF family genes have been reported [13,15], but we still have limited understanding of the molecular functions of most of them. The research described in the current study extends our understanding of the NAC TFs by showing that one member of the family, NAC056, regulates the NO_3_^−^-dependent control of plant root growth. It also plays a wider role by regulating many N-response genes and nitrate assimilation, thus changing plant root growth.

It is well known that one of the primary responses to nitrate is the promotion of LR growth, although how this is achieved is still unclear. Here, our study indicates that nitrate supplementation can clearly induce *NAC056* expression, and that overexpression of *NAC056* promotes LR growth, leading to the conclusion that *NAC056* is involved in the nitrate signal transduction pathway. In addition, the phenotype caused by overexpression of *NAC056* is not limited to root growth. Transcriptome sequencing, combined with RT-qPCR, revealed that several genes encoding nitrate reductase (*NIA*) were identified. Fast and strong induction of *NAC056* by nitrate implies that the NAC proteins are synthesized, but *de novo* protein synthesis *per se* is not required for nitrate induction. The best explanation of the observed results is that *NAC056* is involved in the primary induction of N assimilation by nitrate, leading to promotion of LR growth.

Studies have shown that the plant genome encodes a very large number of NAC TFs. There are at least 106 *NAC*s in the *Arabidopsis* genome [13]. Reflecting the various functions of the NAC TFs, each gene may have its own unique DNA-binding sequence, meaning that each gene participates in multiple signaling pathways to regulate the expression of different target genes. Previous studies indicate that the three stress-induced NAC TFs, namely NAC019, NAC055, and NAC072, bind to the CACG motif [13]. In addition, they also target the sequences CGTG or CACG in the promoter of the NAC genes *RD26*/*NAC072*. *ORE1*, *NAP*, *NTL4* and *NAC017* genes all contain CACG or CGTG sequences [13], whereas NAC013, NAC017, NAC053/NTL4, and NAC078 bind to the CTTGXXXXXCA[AC]G(MDM) domain [17–20]. These results indicate that NAC056 has a close phylogenetic relationship with these NACs. In addition, some reports showed that the JUB1/NAC042-binding motif, TGCCGTXXXXXXXACG, did not contain the CACG core sequence. We used the yeast one-hybrid assay to identify the NAC056BM sequence, T [T/G/A] NCTTG (Fig 6C). Point mutation analysis showed that T (1st) and CTTG (4th to 7th) were critical to the function of the NAC056BM (Fig 6D). Thus, NAC0566BM differs from the previously identified binding motifs for NAC TFs. Different binding motifs specific to particular NACs may have evolved to provide further selectivity in regulating their target genes during plant development, as a result of different internal and external signals. NTL6-binding motif exhibits some sequences with similarity to those of NAC056. However, their gene functions seem to be quite different. NTL6 affects the freezing stress response, and decreased NTL6 activity results in greater susceptibility to pathogen infection at low temperatures.

Many important N-responsive TFs that regulate the expression of nitrogen uptake and assimilation have been identified, for example: CCA1 (Myb TF) and LBD 37/38/39 [33,34]. Rice DST transcription factor is necessary for complete nitrate reductase activity and directly regulates the expression of *OsNR1*.*2* [35]. In addition, NIN-like Protein (NLP) has been well demonstrated to participate in the early N response. NIN-like protein 5 (ZmNLP5) transcription factor is involved in regulating nitrogen response in maize [36]. *Arabidopsis* AtNLP7 binds to the *cis* element in the promoter region of *NIR1* and promotes its transcription. AtNLP7 also binds to many other nitrate-associated genes, including ANR1 and NRT1.1 [37]. The Nitrate-CPK-NLP regulatory network has been discovered as an important part of the growth network [38]. We demonstrate in the current study that NAC056 binds to the NAC056BM sequence in the *NIA1* promoter and up-regulates *NIA1* expression. The regulatory modules involving *NAC056-NIA1* were further confirmed by molecular and biochemical analyses. Because NAC056 and NLP7 regulate common downstream target genes; at the same time, NLP proteins can function as heterodimers by interacting with each other. How they cooperate to regulate plant development in nitrate signaling will be a topic worthy of future research.

Altogether, a model is proposed for *NAC056* with respect to the regulation of nitrate assimilation and root growth (Fig 9D). Under conditions of high nitrate availability, *NAC056* expression level is induced and further promotes nitrate assimilation, leading to enhanced root growth. Therefore, *NAC056* mediates the response of plants to environmental nitrate signals to promote root growth in *Arabidopsis*.

## Materials and methods

### Plant materials and growth conditions

Seeds of the *Arabidopsis* Co-0 ecotype (wild type, WT) were used in the research. *NAC056* T-DNA lines (*NAC056-1*, SALK_137131; *NAC056-2*, SALK_010935), the *NAC025* T-DNA insertion line (SM_3_16875), and the *NAC072* T-DNA insertion line (SALK_072276) were obtained from the Arabidopsis Information Resource (TAIR) or the Nottingham Arabidopsis Stock Centre (NASC). For the constitutive and inducible 35S::*NAC056*, 35S::*NAC056*-HA, 35S:: *NAC056*-GFP, and *NAC056*-GR fusion constructs, PCR fragments containing the entire *NAC056* CDS were amplified, using Arabidopsis cDNA, then sub-cloned into the pHB vector [39], and pK7FWG2 vector [32], respectively, and transformed into *Agrobacterium tumefaciens* GV3101 [40]. Primers are listed in S1 Table. Col-0, mutants, and transgenic plants were grown on either half-strength MS medium, containing 0.9% agar, or nitrogen-deficient half-strength MS medium supplemented with various concentrations of nitrate, in growth chambers in a 16-h/8-h (light/dark) cycle with 60% relative humidity (18–22°C). For stress treatments, seedlings were grown for 7 d on half-strength MS medium and transferred to medium supplemented with 5 mM NO_3_^−^, 1 mM PO_4_^−^, 100 mM NaCl, 200 mM mannitol, 0.2 mM sulfate or 6% (w/v) sucrose, or transferred to freezing stress (−4°C) or heat stress (28°C) conditions for a period of time.

### RNA isolation and RT-qPCR analysis

All steps, including RNA isolation, cDNA synthesis, quality control, primer design, real-time PCR, and data analysis, were performed as previously described [41]. RT-qPCR primers are listed in S2 Table.

### GUS staining

The tissues of pro*NAC056*-GUS transgenic plants were washed with water twice. Then, the samples were immersed in 0.5 mg/mL 5-bromo-4-chloro-3-indolyl β-D-glucuronide stain overnight before incubation at 37°C for 24 h, and then placed in 75% ethanol prior to observation.

### Microscopy

Nuclear localization of the NAC056 fusion protein was observed in the transiently expressed tobacco leaves, using a SP8 LIGHTNING confocal laser scanning microscope (Leica, Germany).

### DEX induction assay

The 10-day-old homozygous 4EnhpNAC056-*NAC056*GR-1 transgenic plants were treated with 30 mM DEX, 30 mM DEX plus 100 mM CHX, or dimethyl sulfoxide (DMSO) (Mock). The DEX was dissolved in DMSO at the same working concentration. The roots were sampled after treatment for fixed periods of time and then immediately immersed in liquid nitrogen prior to RNA isolation. Expression levels of the putative target genes were determined by RT-qPCR analysis.

### Affymetrix ATH1 genechip experiments

Total RNA was prepared from seedlings of Col-0 and the *nac056-2* mutant, using the Qiagen RNeasy Plant Mini Kit. RNA concentration was measured with a NanoDrop ND-1000 UV-Visible spectrophotometer (Thermo Scientific). Subsequent procedures, including quality control of RNA samples, preparation and biotin labeling of cRNA, ATH1 GeneChip hybridization, washing, staining, and scanning, were performed. Processing of the ATH1 GeneChip raw data (CEL files) was performed with open-source ROBIN software (http://mapman.gabipd.org). The robust multichip average (RMA)-normalized log2 signal intensities for all ATH1 probe sets, the log2-fold changes, and adjusted *p* values for probe sets with >2-fold differential signal intensity were compiled. Affymetrix CEL-files were submitted to the NCBI Omnibus database (SUB10367339).

### Genetic ontology (GO) analysis

Genetic ontology (GO) annotations were performed using agriGO v2.051,52 (http:://systemsbiology.cau.edu.cn/agriGOv2/index.php), a public online tool and database (S5 Table). The significance level for GO terms was FDR < 0.05.

### Nitrate reductase activity assay

Seedlings of Col-0 and *nac056* mutant lines were grown for 11 d under control conditions and then exposed to a −4°C environment. Seedlings (100 mg fresh weight, FW) were extracted in 1 mL extraction solution and the homogenate centrifuged at 4,000 × *g* for 10 min. The supernatant was collected and NR activity was measured using an NR Assay Kit (BC0080; SolarBio, Beijing, China).

### Arabidopsis protoplasts isolation

Arabidopsis protoplasts were isolated by employing 14-day-old Arabidopsis seedlings. The isolation method was based on previous literature [42].

### EMSA assays

EMSA assays were performed using the LightShift Chemiluminescent EMSA Kit (Thermo Fisher), according to the manufacturer’s instructions. Briefly, 2 μg of purified His-NAC056 protein (expressed by the pET30a vector) were added to the binding reaction. The binding reactions were allowed to proceed at room temperature for 30 min. The sequences of the complementary oligonucleotides used to generate the biotin-labeled and -unlabeled probes are shown in S3 Table.

### ChIP assays

ChIP assays were performed as described previously [32,42]. The 35S::*NAC056*-HA or Col-0 seedlings were first grown for three weeks at 22°C on half-strength MS medium. Then, the plants were grown in darkness for 2 days at 22°C, fixed with 1% formaldehyde under vacuum for 15 min, then fixation was stopped by the addition of 2 M glycine to a final concentration of 0.15 M, and incubated was continued for another 5 min under vacuum. The chromatin was isolated and sonicated, and DNA fragments associated with the NAC056-HA protein were co -immunoprecipitated using anti-HA antibody (Sigma-Aldrich). The enrichment of DNA fragments was quantified by qPCR using the primers listed in S4 Table.

### Statistical analysis

Student’s *t*-test was used to compare samples, with three independent replicate experiments being conducted. Values are presented as mean ± standard error (SE) or mean ± standard deviation (SD). *p < 0.05 by Student’s *t*-test.

## Supporting information

S1 Fig Phylogenetic tree analysis of class III Subfamily NAC TFs. Phylogenetic tree analysis of class III Subfamily NAC TFs by using DNA MAN 6.0 and MEGA 4.1 software.
(TIF)

S2 Fig Transcriptomic analysis of *LBD16* during LR initiation. After gravitropic stimulation to induce the synchronized initiation of lateral root primordia (LRP) at the surface of bending roots, LRP stages from I to VIII, in accordance with previous reports. *LBD16* gene expression pattern at each time point during LR initiation were measured every 6 hours from 0 to 54 hours post gravitropic induction (pgi). RNA extraction of a population of 5-day-old seedlings bending roots were micro dissected at 10 time points. At least 30 LRP were observed at each time point. The error bars denote SDs. Student’s *t*-test was applied.
(TIF)

S3 Fig Root growth analysis of *NAC025* and *NAC072* gene mutants. A, Relative expression levels of *NAC025* and *NAC072* genes in the mutant and wild-type (Col-0) background. B, Primary root length and (C) LR density analysis in 7-d-old wild-type (Col-0) plants, *nac25-1* (SM_3_16875) and *nac072* (SALK_072276) mutants background. Values are given as mean ± SD, n = 3. *p < 0.05 by student’s *t* test. D, RT-PCR analysis of *NAC056* expression levels in Col-0, *nac056-1* and *nac056-2* background.
(TIF)

S4 Fig Relative GUS activity in root before and after nitrate treatment. The relative GUS activity in proNAC056::GUS plant root before and after 5mM nitrate treatment for 4 hours. *indicates significant differences (*p* < 0.05). The error bars represent SDs (n = 3).
(TIF)

S5 Fig Nitrate induction of *NAC056* expression in NR-null *nia1nia2* mutant background. Nitrate reductase-null plant (*nia1nia2*) was grown in media for one week and then treated with 5 mM KNO_3_ or 5 mM KCl for 0–3 hours. The RNA level of the *NAC056* gene was measured via RT-qPCR. *indicates significant differences (*p* < 0.05). The error bars represent SDs (n = 3).
(TIF)

S6 Fig Sub-localization analysis of NAC056 protein in tobacco leaves. The NAC056 protein has a Nuclear Localization Signal (NLS) between 287 aa-315 aa (http://nls-mapper.iab.keio.ac.jp/cgi-bin/NLS_Mapper_form.cgi). Tobacco leaves were injected with Agrobacterium carrying 35S::GFP and 35S::NAC056-GFP and 35S::NAC056△NLS-GFP (truncated NAC056 protein without the NLS signal) plasmids and then observed using a confocal microscope. The NAC056 protein nucleus localization relies on the NLS signal. The red arrow indicates the nucleus.
(TIF)

S7 Fig The LR phenotype in the *nac056-2* mutant complementary lines. Effects of various concentrations of nitrate (20, 50, and 200 μM) on lateral root growth. WT, *nac056-2* mutant, and the complementary lines were grown vertically in media supplemented with various nitrate concentrations for 7 days. LR density were quantified. LR density was calculated by dividing the LR number per 1 cm of primary root. *indicates significant differences (*p* < 0.05). The error bars represent SDs (n = 3).
(TIF)

S8 Fig Stress-responsive TFs expression levels in Col-0 and *nac056-2* mutant. A, stress response related NAC family genes *NAC019*, *NAC053*, *NAC072*, *NAC096* expression levels in Col-0 and *nac056-2* mutant background. B, Stress-responsive *AREB1*, *DREB1* and *RD29B* expression levels in Col-0 and *nac056-2* mutant background. Values are given as mean ± SD, n = 3. *p<0.05 by student’s *t* test.
(TIF)

S9 Fig Relative *NAC056* expression levels in the *NAC056*-GR transgenic plants before and after dexamethasone (DEX) induction. *NAC056* expression level in *NAC056*-GR transgenic lines after 30 μM DEX induction for a period of time. Values are given as mean ± SD, n = 3.
(TIF)

S10 Fig E.coli Expression and purification of NAC056 protein. A, we used the GenScript GenSmart codon optimization tool (https://www.genscript.com/gensmart-free-gene-codon-optimization.html) to optimize the *NAC056* CDS sequence. Using the pET30a vector and E. coli expression system to express the NAC056 protein. The induction condition is 0.5mM IPTG at 28°C for 4 hours. B, Purification of NAC056 protein using the TaKaRa Capturem™ His-Tagged Purification Kit according to the product instruction.
(TIF)

S11 Fig Expression levels in the wild-type and 35S::*NAC056* transgenic plants. *NAC056* expression level in wild-type (Col-0) and transgenic 35S::*NAC056* transgenic lines. Values are given as mean ± SD, n = 3.
(TIF)

S1 Table The primers used in vector construction and mutant analysis. (DOCX)

S2 Table Gene-specific primers used in the qRT-PCR experiments. (DOCX)

S3 Table In vitro synthesized probe sequences for EMSA analysis. (DOCX)

S4 Table Gene-specific primers used in the ChIP-qPCR experiments. (DOCX)

S5 Table The source of GO annotations in Fig 6B. (XLS)
