# Supplementary figures and images for: The nitrate-inducible NAC transcription factor NAC056 controls nitrate assimilation and promotes lateral root growth in Arabidopsis thaliana
Source: PLoS Genet. 2022 Mar 9;18(3):e1010090. doi: 10.1371/journal.pgen.1010090 (PMC8989337; doi:10.1371/journal.pgen.1010090)

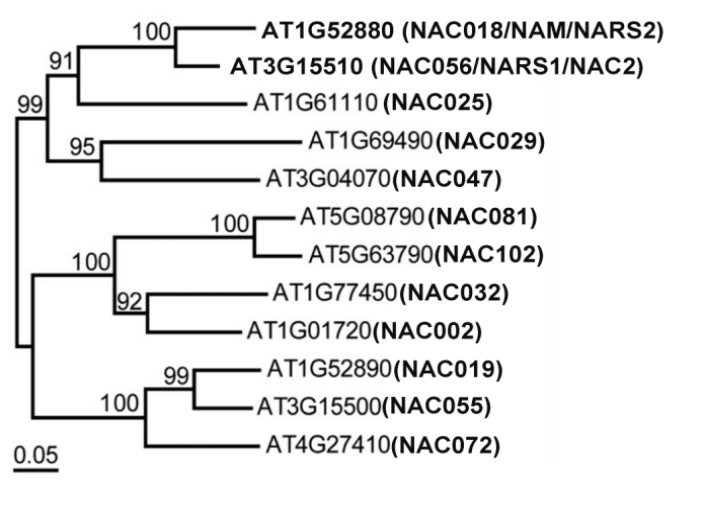

Supplement: S1 Fig — Phylogenetic tree analysis of class III Subfamily NAC TFs by using DNA MAN 6.0 and MEGA 4.1 software. (TIF) [file pgen.1010090.s001.tif]

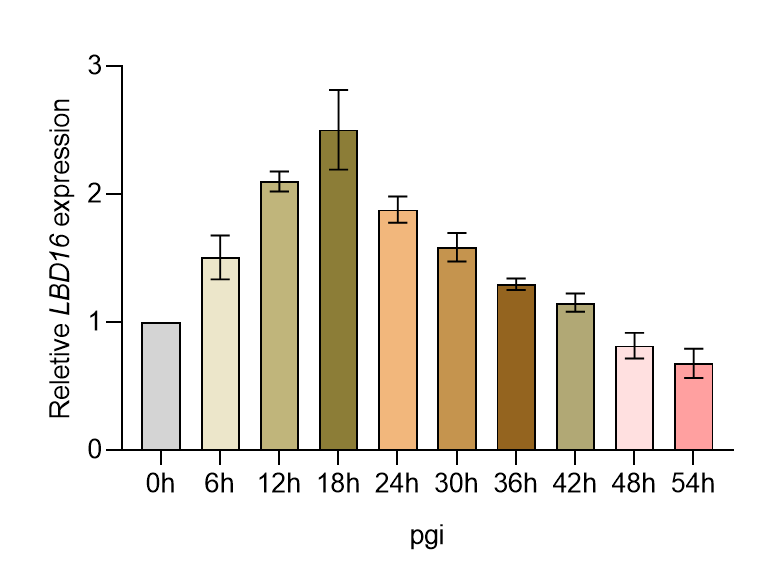

Supplement: S2 Fig — After gravitropic stimulation to induce the synchronized initiation of lateral root primordia (LRP) at the surface of bending roots, LRP stages from I to VIII, in accordance with previous reports. LBD16 gene expression pattern at each time point during LR initiation were measured every 6 hours from 0 to 54 hours post gravitropic induction (pgi). RNA extraction of a population of 5-day-old seedlings bending roots were micro dissected at 10 time points. At least 30 LRP were observed at each time point. The error bars denote SDs. Student’s t-test was applied. (TIF) [file pgen.1010090.s002.tif]

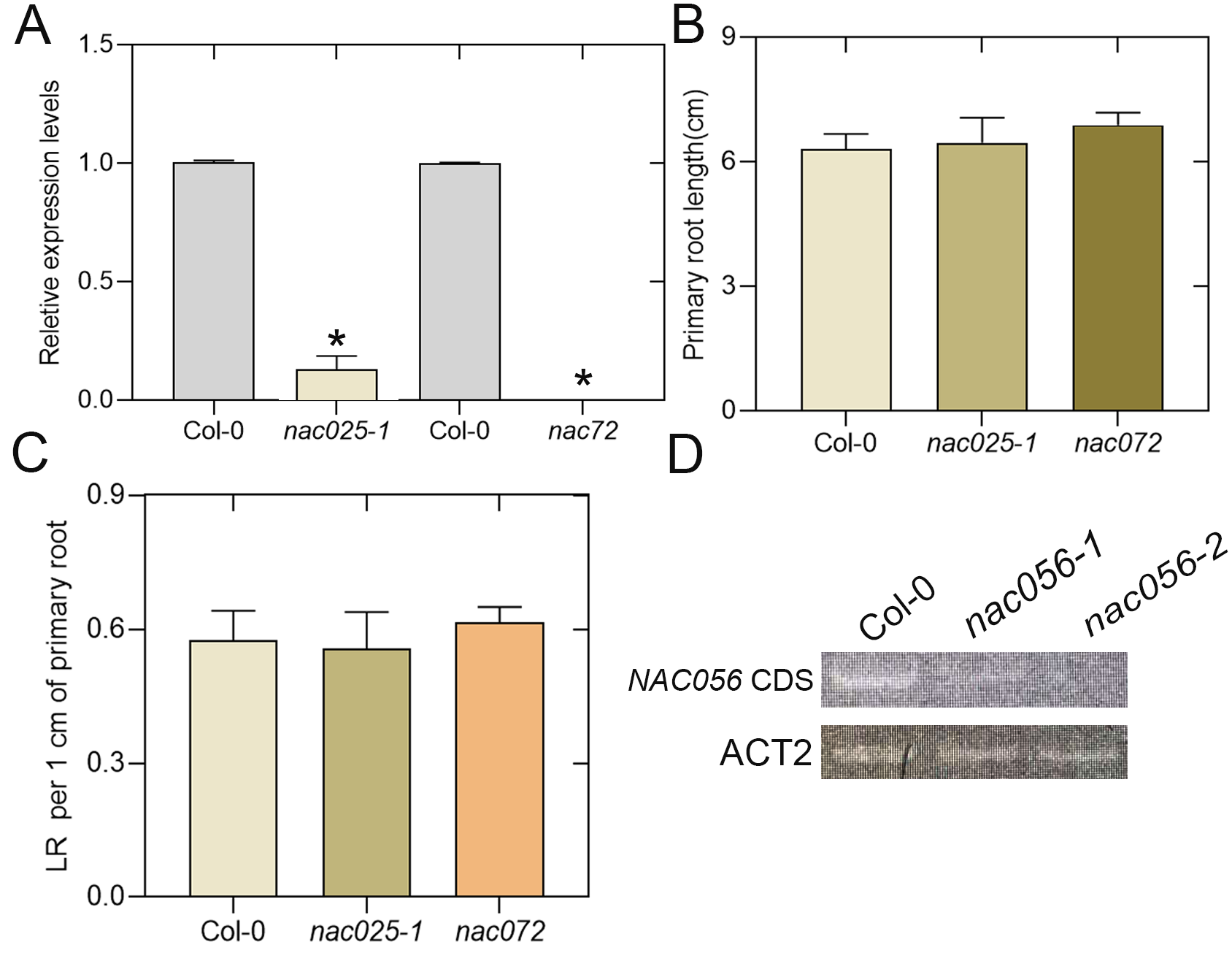

Supplement: S3 Fig — A, Relative expression levels of NAC025 and NAC072 genes in the mutant and wild-type (Col-0) background. B, Primary root length and (C) LR density analysis in 7-d-old wild-type (Col-0) plants, nac25-1 (SM_3_16875) and nac072 (SALK_072276) mutants background. Values are given as mean ± SD, n = 3. *p < 0.05 by student’s t test. D, RT-PCR analysis of NAC056 expression levels in Col-0, nac056-1 and nac056-2 background. (TIF) [file pgen.1010090.s003.tif]

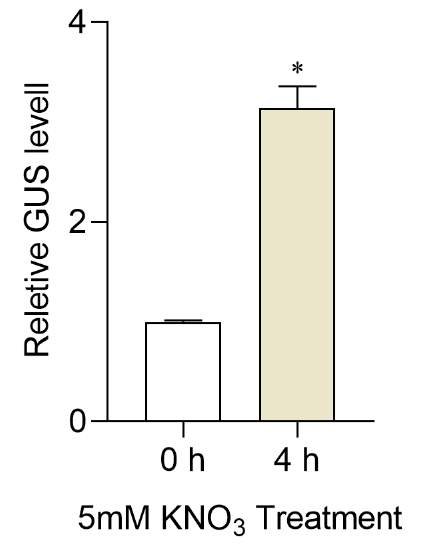

Supplement: S4 Fig — The relative GUS activity in proNAC056::GUS plant root before and after 5mM nitrate treatment for 4 hours. *indicates significant differences (p < 0.05). The error bars represent SDs (n = 3). (TIF) [file pgen.1010090.s004.tif]

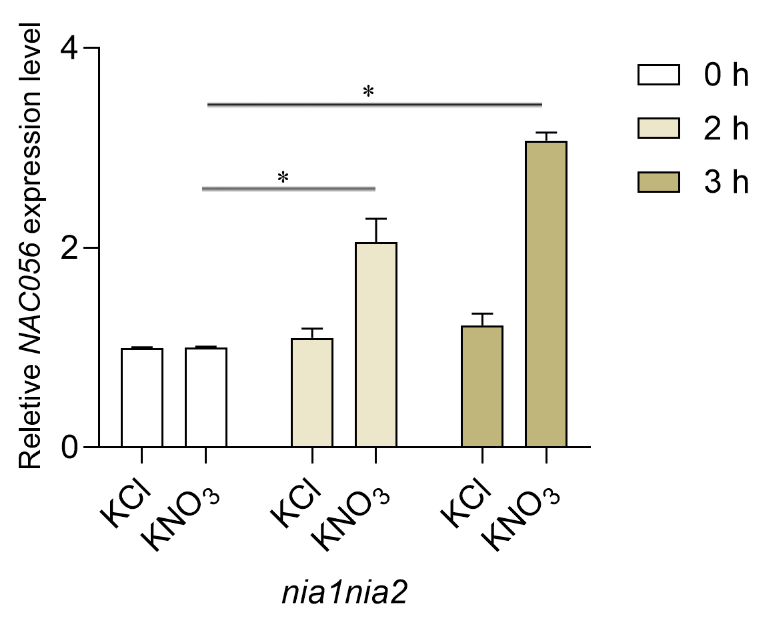

Supplement: S5 Fig — Nitrate reductase-null plant (nia1nia2) was grown in media for one week and then treated with 5 mM KNO3 or 5 mM KCl for 0–3 hours. The RNA level of the NAC056 gene was measured via RT-qPCR. *indicates significant differences (p < 0.05). The error bars represent SDs (n = 3). (TIF) [file pgen.1010090.s005.tif]

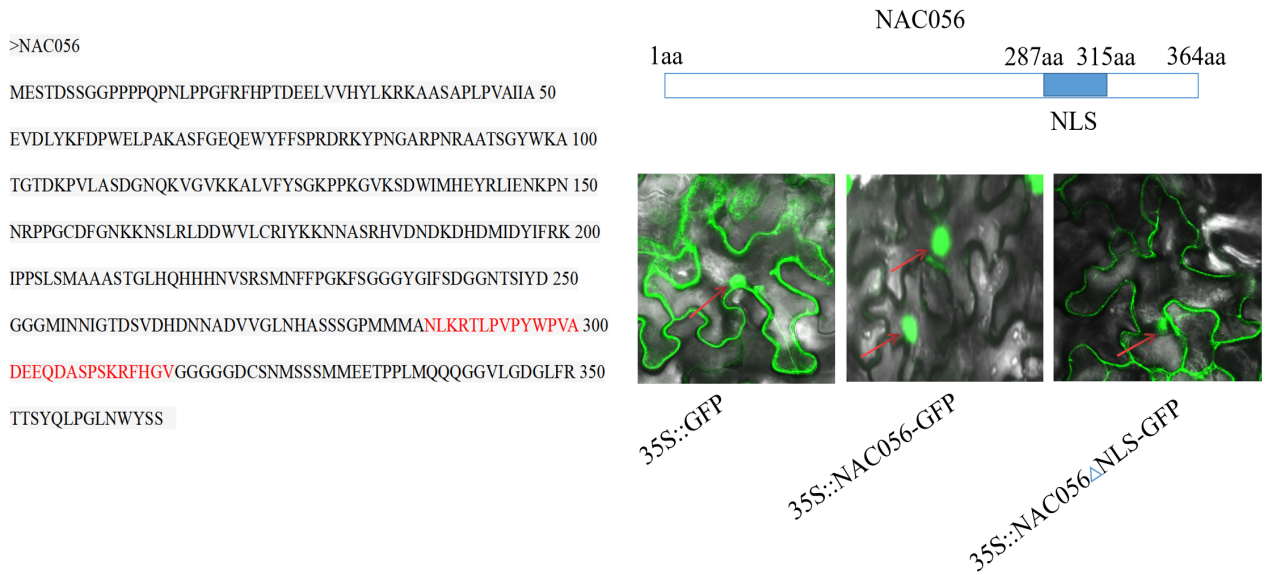

Supplement: S6 Fig — The NAC056 protein has a Nuclear Localization Signal (NLS) between 287 aa-315 aa (http://nls-mapper.iab.keio.ac.jp/cgi-bin/NLS_Mapper_form.cgi). Tobacco leaves were injected with Agrobacterium carrying 35S::GFP and 35S::NAC056-GFP and 35S::NAC056△NLS-GFP (truncated NAC056 protein without the NLS signal) plasmids and then observed using a confocal microscope. The NAC056 protein nucleus localization relies on the NLS signal. The red arrow indicates the nucleus. (TIF) [file pgen.1010090.s006.tif]

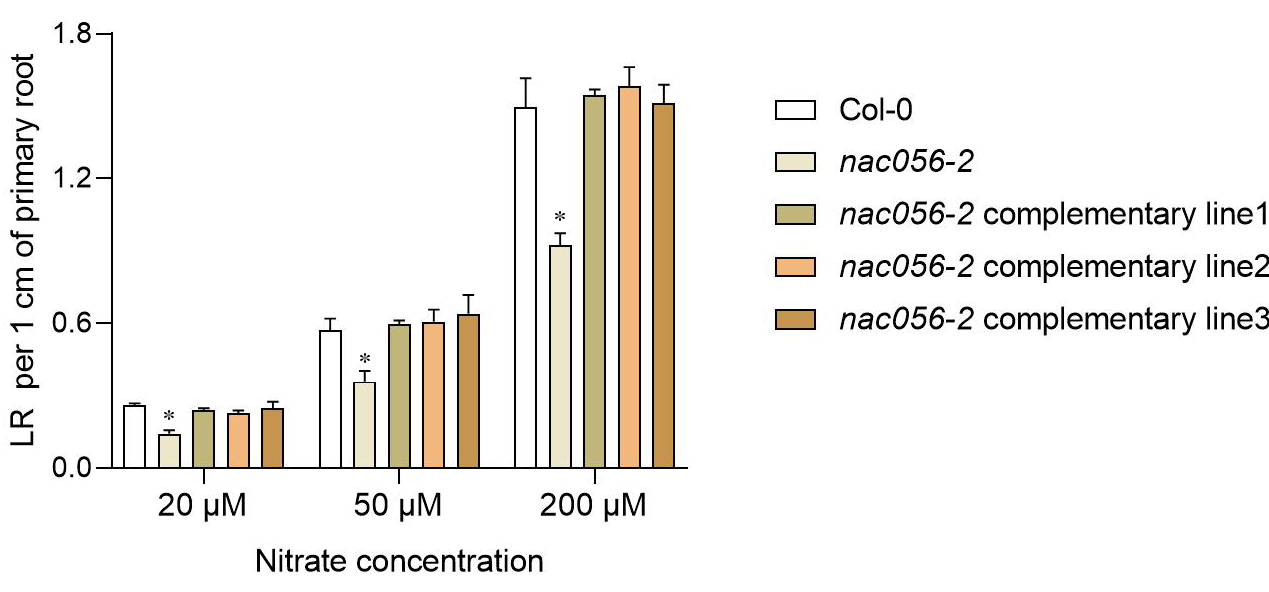

Supplement: S7 Fig — Effects of various concentrations of nitrate (20, 50, and 200 μM) on lateral root growth. WT, nac056-2 mutant, and the complementary lines were grown vertically in media supplemented with various nitrate concentrations for 7 days. LR density were quantified. LR density was calculated by dividing the LR number per 1 cm of primary root. *indicates significant differences (p < 0.05). The error bars represent SDs (n = 3). (TIF) [file pgen.1010090.s007.tif]

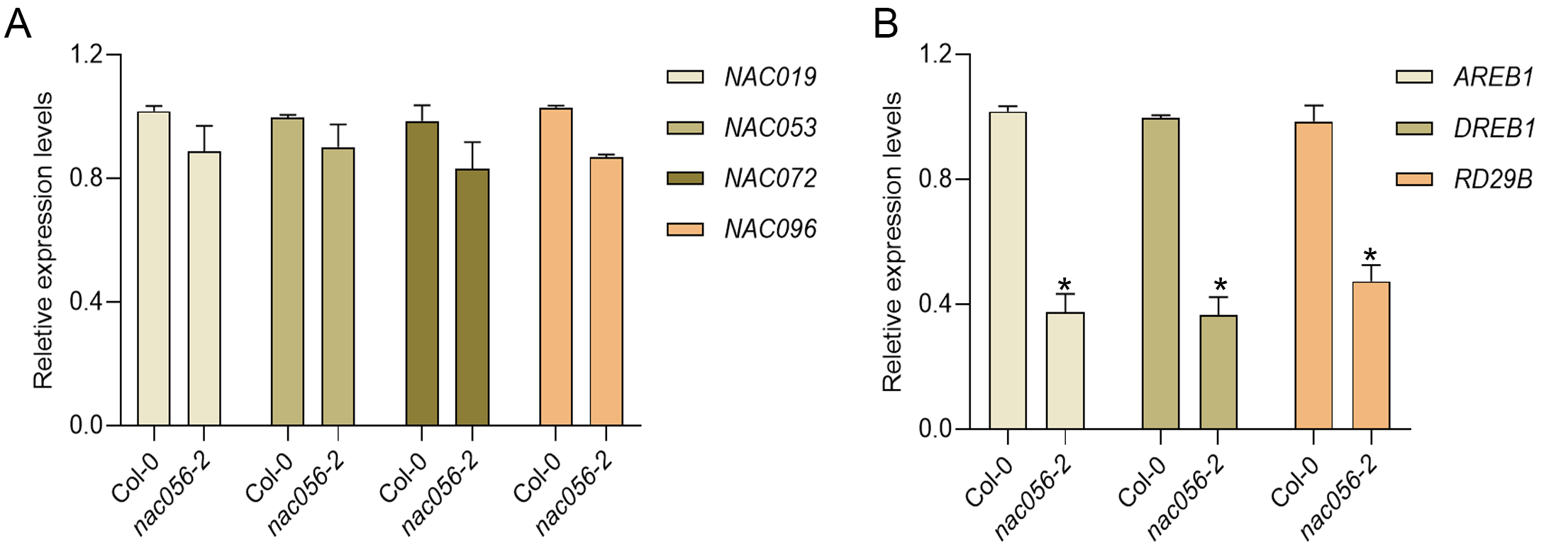

Supplement: S8 Fig — A, stress response related NAC family genes NAC019, NAC053, NAC072, NAC096 expression levels in Col-0 and nac056-2 mutant background. B, Stress-responsive AREB1, DREB1 and RD29B expression levels in Col-0 and nac056-2 mutant background. Values are given as mean ± SD, n = 3. *p<0.05 by student’s t test. (TIF) [file pgen.1010090.s008.tif]

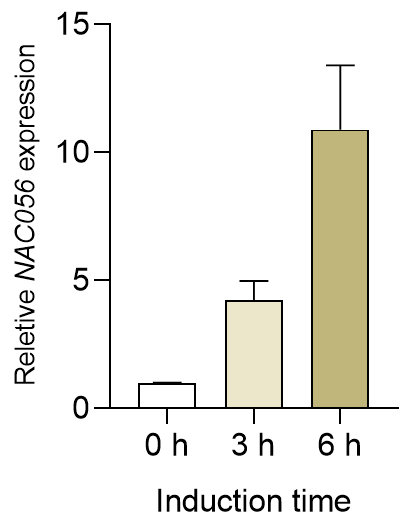

Supplement: S9 Fig — NAC056 expression level in NAC056-GR transgenic lines after 30 μM DEX induction for a period of time. Values are given as mean ± SD, n = 3. (TIF) [file pgen.1010090.s009.tif]

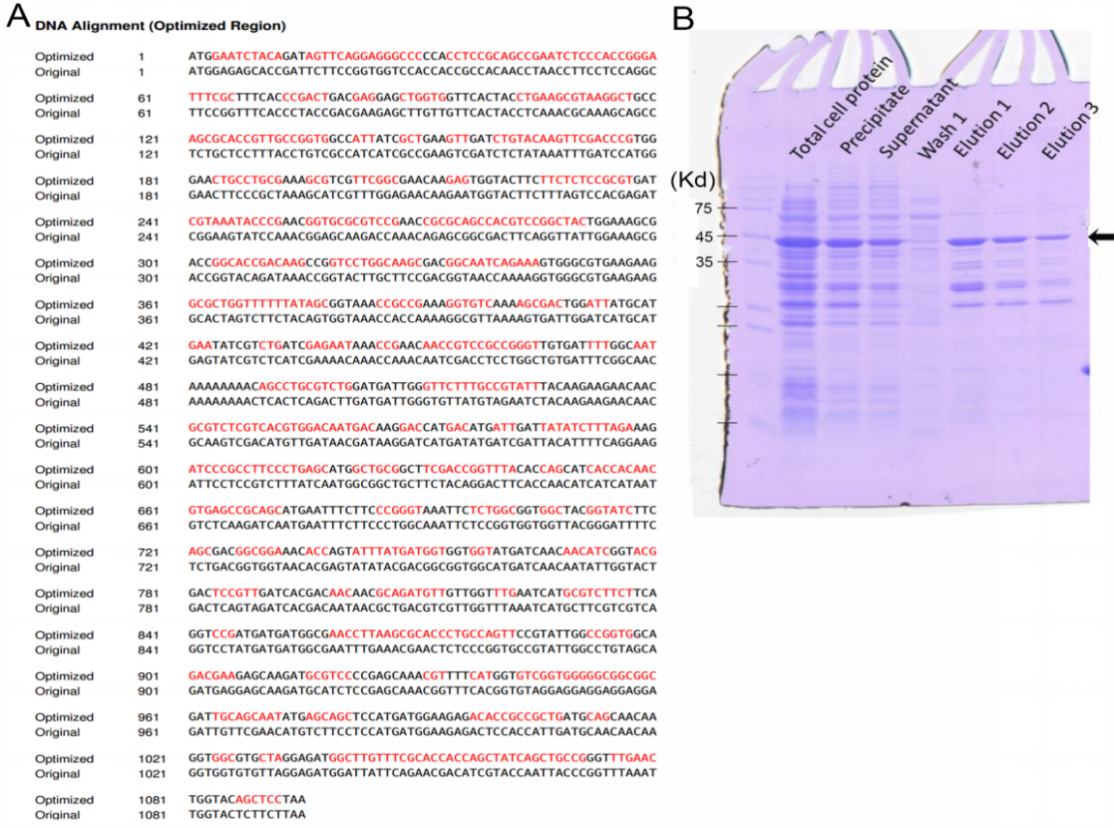

Supplement: S10 Fig — A, we used the GenScript GenSmart codon optimization tool (https://www.genscript.com/gensmart-free-gene-codon-optimization.html) to optimize the NAC056 CDS sequence. Using the pET30a vector and E. coli expression system to express the NAC056 protein. The induction condition is 0.5mM IPTG at 28°C for 4 hours. B, Purification of NAC056 protein using the TaKaRa Capturem™ His-Tagged Purification Kit according to the product instruction. (TIF) [file pgen.1010090.s010.tif]

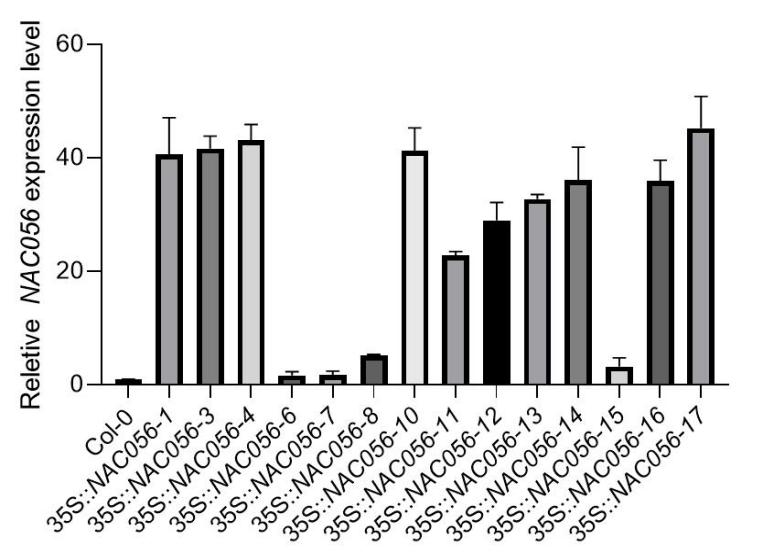

Supplement: S11 Fig — NAC056 expression level in wild-type (Col-0) and transgenic 35S::NAC056 transgenic lines. Values are given as mean ± SD, n = 3. (TIF) [file pgen.1010090.s011.tif]
