# Supplementary material for: The nitrate-inducible NAC transcription factor NAC056 controls nitrate assimilation and promotes lateral root growth in Arabidopsis thaliana
Source: PLoS Genet. 2022 Mar 9;18(3):e1010090. doi: 10.1371/journal.pgen.1010090 (PMC8989337; doi:10.1371/journal.pgen.1010090)
Supplement: S1 Table — (DOCX) [file pgen.1010090.s012.docx]

**S1_Table. The primers used in vector construction and mutant analysis.**

| Transgenes | Primers (Sequence 5’-3’) |
| --- | --- |
| Clone *NAC056* cDNA | 5'- ATGGAGAGCACCGATTCTTCCGGT-3′^a^ |
|  | 5'-TTAAGAAGAGTACCAATTTAAACC-3′^a^ |
| pCAMBIA1300::*NAC056* | 5'-CTGCAG ACATTGAAAAGTCGAGTTTACGA-3′^b^ |
|  | 5'-GGGCCC TGTATCCGTTGTAGAAGATATCTA-3′^b^ |
| Double 35S::*NAC056* | 5'-GGATCC ATGGAGAGCACCGATTCTTCCGGT-3′ |
|  | 5'-CTAGAA TTAAGAAGAGTACCAATTTAAACC-3′ |
| pER8::*NAC056* | 5'-ACTAGT ATGGAGAGCACCGATTCTTCCGGT-3′ |
|  | 5'-CTCGAG TTAAGAAGAGTACCAATTTAAACC-3′ |
| pER8::*NAC056*-HA | 5'-ACTAGT ATGGCGACTC AAGATTCTCA AGG -3′ |
|  | 5'-GAGCTC TTAAGAAGAGTACCAATTTAAACC-3′ |
| pET30a::*NAC056* | 5'-GAATTC ATGGTTTCTCTTCTTACAATGC -3′ |
|  | 5'-GGATCC TTATAATTGATCAACGAGTTCAT -3′ |
| SALK_137131 | LP GGCACTGCGTCGTTATATAGG |
|  | RP AGACTCCACCATTGATGCAAC |
| SALK_035935 | LP AGAAGAACAACGCAAGTCGAC |
|  | RP CCATCGACTATTTCGCCTTAAC |
| SM_3_16875 | LP GTAATTTCCTCGTCGAGGGAC |
|  | RP TGAAGAAACGACCATACCGTC |
| SALK_072276 | LP TTAATGCATGTGTGGGTGATG |
|  | RP CCCGGAGATAAAAGACCAGTC |

Restriction digestion sites are underlined.

a: Primers designed according to the 5’/3’-UTR for clone *NAC056* gene cDNA.

b: Primers for constitutive overexpression of the *NAC056* gene
