# Supplementary material for: The nitrate-inducible NAC transcription factor NAC056 controls nitrate assimilation and promotes lateral root growth in Arabidopsis thaliana
Source: PLoS Genet. 2022 Mar 9;18(3):e1010090. doi: 10.1371/journal.pgen.1010090 (PMC8989337; doi:10.1371/journal.pgen.1010090)
Supplement: S2 Table — (DOCX) [file pgen.1010090.s013.docx]

**S2_Table. Gene-specific primers used in the qRT-PCR experiments.**

| Genes | Primers (Sequence 5’-3’) |
| --- | --- |
| *AtACT2* | 5’-TCAGATGCCCAGAAGTCTTGTT-3’ |
|  | 5’-CCGTACAGATCCTTCCTGATATC-3’ |
| *AtTUB2* | 5’-GAGCCTTACAACGCTACTCTGTC-3' |
|  | 5’-ACACCAGACATAGTAGCAGAAA-3' |
| *NAC056* | 5’-ATTCTCCGGTGGTGGTTACG -3' |
|  | 5’-GACCTGACGACGAAGCATGA-3' |
| *NAC018* | 5’-CCGCGAAAGCTTCGTTTGGA-3' |
|  | 5’-GTCGCAGCTCGGTTAGGTCT-3' |
| *NAC025* | 5’-AGCTTCCAAGCAAGGCGAGT-3' |
|  | 5’-GGAAGTTGCTGCCCGGTTTG-3' |
| *NAC029* | 5’-GACGTGTTCGCTGGCTCATT -3' |
|  | 5’-CCCCGAACCAACTAGACTCCG-3' |
| *NAC047* | 5’-CTCTTCCCCAGTCCCGCTTT-3' |
|  | 5’-CCCAAATGGAGCCTTAGCTGG-3' |
| *NAC081* | 5’-TTGGAGGTTCAGAGCGAGCC-3' |
|  | 5’-AAGCGTCAGGCTGCAACAAC-3' |
| *NAC102* | 5'-GCAGCTGGAACCGGTTATTGG-3′ |
|  | 5'-CCCTTTCGGAGCTTTTCCTGC-3′ |
| *NAC019* | 5'-CTCGGTACCGGAACTCGGAA-3' |
|  | 5'-CCCGTGACTGCTCTCGACTT-3' |
| *NAC055* | 5'-AGAGCTTGACCCGTTAGCCC-3' |
|  | 5'-GTGACCGGCGGCTTTTCTAC-3' |
| *NAC072* | 5'-ATAGAGTAGCCGGGTCGGGT-3' |
|  | 5'-TTGGGAGCTTTTCCGGCGTA-3' |
| *NAC032* | 5'-CGGTCCGCTGGTTCTGGTTA-3' |
|  | 5'-CTCCCTTTGGAGCTTTGCCG-3' |
| *NAC002* | 5'-TTCGACACGTCGGATTCGGT-3' |
|  | 5'-TTGGGCTCGCTCTGAACCTC-3' |
| *NIA1* | 5'-ATCCATGCTCCGTCTCACCG-3' |
|  | 5'-CGTGGTTGCGGACGTAATGG-3' |
| *NIA2* | 5'-AGCTGGATCGAGCGTAACCC-3' |
|  | 5'-ACGAACGTAGTGCAACGGGA-3' |
| *NRT1.1* | 5'-ACGTCGTCTCACTGCGAACA-3' |
|  | 5'-CACGCCTCCCGTTCCTAGAG-3' |
| *NRT1.2* | 5'-TCAAGCAATGCGGTTGCGAG-3' |
|  | 5'-TTAGTTGTGCCCGAGGAGGC-3' |
| *NRT1.6* | 5'-TTGGAGGCTGGAGAGCCATT-3' |
|  | 5'-AGCTTCTACTGGCTCCATGTG-3' |
| *NRT2.1* | 5'-TGCCTCCTTCCACAGATCCA-3' |
|  | 5'-GCAAACCGGAGGCTTCCTTG-3' |
| *NRT2.2* | 5'-TTGCTTGCACGTTGCCTGTT-3' |
|  | \|  \| 5'- TCCGTAGCGTCGACCGAATC-3' \| \| --- \| --- \| |
| *NRT3.1* | 5'-TTGCCTATGGACAGAGCACCG-3' |
|  | 5'-GAGCCACGACGGAGAAGACA-3' |
| *CBF1* | 5'-TCCAAACCGCTGAGATGGCA -3' |
|  | 5'-CTTGGCGCATGTTGACTCCG-3' |
| *CBF2* | 5'-ATACGCCGGAACAGAGCCAA-3' |
|  | 5'-CGGCGACGGTAAAAGCATCC-3' |
| *CBF3* | 5'-GCTGACTCGGCTTGGAGACT-3' |
|  | 5'-TCCGTCGTCGCATCACACATA-3' |
