# Supplementary material for: The nitrate-inducible NAC transcription factor NAC056 controls nitrate assimilation and promotes lateral root growth in Arabidopsis thaliana
Source: PLoS Genet. 2022 Mar 9;18(3):e1010090. doi: 10.1371/journal.pgen.1010090 (PMC8989337; doi:10.1371/journal.pgen.1010090)
Supplement: S3 Table — (DOCX) [file pgen.1010090.s014.docx]

**S3_Table. In vitro synthesized probe sequences for EMSA analysis.**

NIA1-Motif a:

TTTTATCCATTTTTCTTGGATTTTTTTGTGGGTTCATTTTGGTAGTTCGGGTATATAGTTCACTTTAACATGACTTGCAAGATAATGCGCTACTGA

NIA1-Motif b:

GAAAATTCAAACAAGTGACAAAAATAATATTACGACCCGTCCCTTTGTTTGGTTGCCCCGTTCGTGTATTTGCTTGGCCGTTGGTGTT

NIA1-Motif c:

GATCCAAGGGAAGGTTTATCTCAGCTTTTTTTCCTCAAGAGAAAGGTGTATCTTGCCCGTGGTTACTCGATTGTTGTTCGTATTCAATTTATTAAATTTTATAGCTTCAAGGGAAG

NIA2-Motif d:

GTCAACAACTACGAGCTAAATAATCAAGTTAGGCGATCAATTGAGGTAAAATATCTCACAAGT

NIA2-Motif e:

TATTGGTTACCCAAAATTAGAAGTTCACAAGAAAATCAATATTCAATATTTACTCAATGAAA

NIA2-Motif f:

ATACATAGGTATTTTAAACTTTTAGATGATTCAAGGAACTATTTTAGGTTATAATAATGTATACAAAA

NIA2-Motif g:

AGTCAAGTCATAAGAAAAAATTAATAGTAAGTGTGTAAGAAAAAAATTAAAAAGTCACAAATGGTCCCATACGTGTGATTCGGCACGATACTTCCTAAAAGCATACCACTAGGCTCTCTCCTATATAACTTCATCTCCACCAACGATGCATTTTCGTCCTCAGATCTCATCTCACTCACAAGAGAG
