# Supplementary material for: The nitrate-inducible NAC transcription factor NAC056 controls nitrate assimilation and promotes lateral root growth in Arabidopsis thaliana
Source: PLoS Genet. 2022 Mar 9;18(3):e1010090. doi: 10.1371/journal.pgen.1010090 (PMC8989337; doi:10.1371/journal.pgen.1010090)
Supplement: S4 Table — (DOCX) [file pgen.1010090.s015.docx]

**S4_Table. Gene-specific primers used in the ChIP-qPCR experiments.**

| Transgenes | Primers (Sequence 5’-3’) |
| --- | --- |
| *NIA1*-a | 5’-ATTTTGAGGTCACGATTTTGGT-3' |
|  | 5’- ATCATGTCATAACGACATTTTC-3' |
| *NIA1*-b | 5’- ACAAAACAGAAAATCGAAAAT-3' |
|  | 5’- AAGACTTTGGAGGCTAAGTGG-3' |
| *NIA1*-c | 5’- CTTATACAAGAGTCCATGTTTGT-3' |
|  | 5’-ATTGCACTTAAAAAAGAAGAAA-3' |
| *NIA2*-d | 5’- GAGGATTAATGACGTTATGAC-3' |
|  | 5’- TCATTAAAAAATGAGTCGTCA-3' |
| *NIA2*-e | 5’-ATAAAGATGTATATTGGTTACCC-3' |
|  | 5’- GTTCAGCAGATTCAGTGGGTAA-3' |
| *NIA2*-f | 5’- AACATTCATACATAGGTATTTTA-3' |
|  | 5’-TTAACAGATGTGATTTTCAGCAATA-3' |
| *NIA2*-g | 5’- AACAAATGGATGGTTTATTTAATT-3' |
|  | 5’- GTAGAAAGCAGAGCCTTTTTCTCTC-3' |
